# *Caspase-8*, association with Alzheimer’s Disease and functional analysis of rare variants

**DOI:** 10.1371/journal.pone.0185777

**Published:** 2017-10-06

**Authors:** Jan Rehker, Johanna Rodhe, Ryan R. Nesbitt, Evan A. Boyle, Beth K. Martin, Jenny Lord, Ilker Karaca, Adam Naj, Frank Jessen, Seppo Helisalmi, Hilkka Soininen, Mikko Hiltunen, Alfredo Ramirez, Martin Scherer, Lindsay A. Farrer, Jonathan L. Haines, Margaret A. Pericak-Vance, Wendy H. Raskind, Carlos Cruchaga, Gerard D. Schellenberg, Bertrand Joseph, Zoran Brkanac

**Affiliations:** 1 Department of Psychiatry and Behavioral Sciences, University of Washington, Seattle, WA, United States of America; 2 Department of Oncology-Pathology, Cancer Centrum Karolinska, Karolinska Institutet, Stockholm, Sweden; 3 Department of Genetics, Stanford University, CA, United States of America; 4 Department of Genome Sciences, University of Washington, Seattle, WA, United States of America; 5 Department of Psychiatry, Washington University, St. Louis, MO, United States of America; 6 Department of Psychiatry and Psychotherapy, University of Bonn, Bonn, Germany; 7 Department of Biostatistics and Epidemiology, University of Pennsylvania Perelman School of Medicine, Philadelphia, PA, United States of America; 8 Department of Psychiatry and Psychotherapy, University of Cologne, Cologne, Germany; 9 German Center for Neurodegenerative Diseases, Bonn, Germany; 10 Institute of Clinical Medicine–Neurology, University of Eastern Finland, Kuopio, Finland; 11 Department of Neurology, Kuopio University Hospital, Kuopio, Finland; 12 Institute of Biomedicine, University of Eastern Finland, Kuopio, Finland; 13 Institute of Human Genetics, University of Bonn, Bonn, Germany; 14 Department of Primary Medical Care, University Medical Centre Hamburg-Eppendorf, Hamburg, Germany; 15 Departments of Medicine (Biomedical Genetics), Neurology, Ophthalmology, Epidemiology, and Biostatistics, Boston University, Boston, MA, United States of America; 16 Department of Epidemiology and Biostatistics, Case Western Reserve University, Cleveland, OH, United States of America; 17 Institute for Computational Biology, Case Western Reserve University, Cleveland, OH, United States of America; 18 The John P. Hussman Institute for Human Genomics, University of Miami, Miami, FL, United States of America; 19 Dr. John T. Macdonald Foundation Department of Human Genetics, University of Miami, Miami, FL, United States of America; 20 Department of Medicine, University of Washington, Seattle, WA, United States of America; 21 Department of Pathology and Laboratory Medicine, University of Pennsylvania Perelman School of Medicine, Philadelphia, PA, United States of America; Torrey Pines Institute for Molecular Studies, UNITED STATES

## Abstract

The accumulation of amyloid beta (Aβ) peptide (Amyloid cascade hypothesis), an APP protein cleavage product, is a leading hypothesis in the etiology of Alzheimer's disease (AD). In order to identify additional AD risk genes, we performed targeted sequencing and rare variant burden association study for nine candidate genes involved in the amyloid metabolism in 1886 AD cases and 1700 controls. We identified a significant variant burden association for the gene encoding caspase-8, *CASP8* (p = 8.6x10^-5^). For two CASP8 variants, p.K148R and p.I298V, the association remained significant in a combined sample of 10,820 cases and 8,881 controls. For both variants we performed bioinformatics structural, expression and enzymatic activity studies and obtained evidence for loss of function effects. In addition to their role in amyloid processing, caspase-8 and its downstream effector caspase-3 are involved in synaptic plasticity, learning, memory and control of microglia pro-inflammatory activation and associated neurotoxicity, indicating additional mechanisms that might contribute to AD. As caspase inhibition has been proposed as a mechanism for AD treatment, our finding that AD-associated CASP8 variants reduce caspase function calls for caution and is an impetus for further studies on the role of caspases in AD and other neurodegenerative diseases.

## Introduction

Alzheimer’s Disease (AD) is the most common form of dementia and with general ageing of the population, the incidence and prevalence of AD have been dramatically rising [1]. The neuropathologic finding that amyloid beta (Aβ) peptide, an APP protein cleavage product, is a component of amyloid plaques [2] and the observation that mutations in *APP*, and genes that affect APP cleavage: *PSEN1* and *PSEN2*, [3–5], cause early onset AD suggested that APP plays a particularly important role in AD pathogenesis [6–9] and were the basis for the amyloid cascade hypothesis of AD [10–12]. According to this hypothesis, dysregulation of APP metabolism is the key event in the development of AD, which sequentially leads to aggregation of β-amyloid, development of neurofibrillary tangles, disruption of synaptic connections, and eventually neuronal death that ultimately manifests clinically as dementia. The amyloid cascade hypothesis spurred us to evaluate genes that might be involved in the APP metabolism for contribution to risk for AD.

Based on a literature survey, we selected nine genes involved in the amyloid metabolism and involved in APP cleavage to test for the association with AD. We chose *APH1A*, *APH1B*, *NCSTN* and *PSENEN* [13] as they are members of γ-secretase complex that cleaves APP, as well as β-secretase component BACE1 [14]. GSK3A and GSK3B were selected for their involvement in Aβ production regulation through phosphorylation of APP and γ-secretase complex proteins [15]. We selected *CASP3* and *CASP8* given the evidence that they are able to cleave APP [16]. Caspases lead to elevated β-amyloid levels during apoptosis which can be reduced down to and below normal levels by caspase inhibition [17]. On the other hand Aβ is neurotoxic and can initiate apoptosis [18] which might lead to a positive feedback loop.

To our knowledge most of the subjects in our discovery cohort were not screened for mutations in *APP*, *PSEN1* or *PSEN2* and *TREM2* was only recently identified [19]. Thus we have included *APP*, *PSEN1*, *PSEN2* and *TREM2* in our study to identify subjects where variants in these genes might be causal and to assess the validity of our approach. The inclusion of these known AD contributing genes where multiple rare alleles contribute to risk, should also allow us to empirically test if our sequencing based approach is sufficiently powered to detect associations of known AD genes and thus if it is suitable for new gene detection. To evaluate the association of amyloid metabolism genes with AD we used targeted sequencing and variant-burden association analysis of rare, protein disrupting (canonical splice, truncating, stop) and missense variants. After identifying a statistically significant variant-burden association with *CASP8*, for two missense variants, K148R and I298V we performed a replication study to confirm the association and functional studies to evaluate how the identified variants affect caspase function.

## Methods

### Ethics statement

All procedures performed in studies involving human participants were in accordance with the ethical standards of the institutional and/or national research committee and with the 1964 Helsinki declaration and its later amendments or comparable ethical standards. This article does not contain any studies with animals performed by any of the authors. Prior to commencement the study was reviewed and approved by the University of Washington Institution Review Board (IRB#33186). Informed consent was obtained from all individual participants included in the study.

### Subjects

Subjects in targeted sequencing and variant-burden association study were of European American descent. Samples were provided by NIA-LOAD, NCRAD, NACC, NIMH, ACT and Washington University (WashU). Replication studies on *CASP8* variants K148R and I298V were performed with subjects from Finland, Germany and the Alzheimer's Disease Genetics Consortium (ADGC). Detailed information about subjects is presented in supplementary materials, **S1 File**.

### Sequencing, genotyping and variant annotation

We employed molecular inversion probes (MIPs) [20] for targeted capture of coding exons of selected genes. Following target capture, samples were individually barcoded and pooled 192 at a time for sequencing on an Illumina Hiseq 2000/2500 instrument. Reads were aligned with BWA [21]. MIP-arms were removed and overlapping regions of the two sequences of a single read were reduced to one sequence per MIP region. Duplicates were removed based on sequence tags with Prinseq [22]. Variants were called with GATK unified genotyper, coverage was determined with the GATK DepthOfCoverage tool. Variants were annotated for Seattleseq 137 annotation pipeline [23] and frequency in the 1000genome dataset. Replication studies of p.K148R and p.I298V *CASP8* variants were conducted by genotyping with Sequenom iPlexa and TaqMan® SNP genotyping assay in Finnish and Germans samples respectively and with the Infinium HumanExome Beadchip for ADGC samples.

Detailed information about sequencing and genotyping can be found in supplementary materials, **S1 File**.

### Association analysis

Burden test of association was performed using a custom script in Python/R. In burden analysis we aggregated all protein disrupting (start/stop variants, canonical splice sites and frameshift) and missense variants with minor allele frequency (MAF) ≤ 0.01 in the 1000 Genomes project for individuals of European ancestry (phase1_release_v3.20101123). Variants were only used in the analysis, if the respecting sites were called in ≥ 95% of cases and controls. Significance for the variant-burden association was determined using one-sided Fisher’s exact tests. The one sided Fisher’s exact test is appropriate for our prior specified hypothesis that rare disruptive and missense variants are causal while logistic regression models might not when the number of subjects with the variant is small [24,25]. Single variant association was performed using one-sided Fisher's test as well.

### Structural modeling of caspase-8 domains

The modeled structure of the DED_2_ and p18 domains of caspase-8 were generated by Phyre2 web portal for protein modeling, prediction and analysis [26]. Met^1^-Asp^177^ and Ser^202^-Asp ^359^ aa sequences of caspase-8 were submitted to the Phyre^2^ server, and the structures resulting from the analysis used as 3D models of caspase-8 2 and caspase-8 p18, respectively. 153 residues (97% coverage of the submitted Ser^202^-Asp^359^ sequence) and 176 residues (99% coverage of the submitted Met^1^-Asp^177^ sequence) were modelled with 100% confidence. Models were depicted in the JSmol molecular viewer.

### Plasmids and site directed mutagenesis

The pcDNA3-Casp8 plasmid encoding the human 479 aa procaspase-8 isoform was obtained from the Addgene plasmid repository (#11817) [27]. The pcDNA3-Casp8 plasmid, hereafter referred as Casp8 WT, was used as a template to generate plasmids encoding the naturally occurring caspase-8 variants K148R and I298V, having amino acid substitutions at residue 148 in the second death-effector domain (DED) within the prodomain or residue 298 in the p18 enzyme subunit of caspase-8 respectively. Site directed mutagenesis was performed with Quick Change II Site-Directed Mutagenesis Kit (#20052, Agilent Technologies) according to the manufacturer’s protocol using primers (5’-GGATATTTTCATAGAGATGGAGAGGAGGGTCATCCTGGGAG-3’ [forward]; 5’-CTCCCAGGATGACCCTCCTCTCCATCTCTATGAAAATATCC-3’ [reverse]) for generation of the pcDNA3-Casp8-K148R plasmid and (5’-CAGTAGAGCAAATCTATGAGATTTTGAAAGTCTACCAACTCATGG-3’ [forward]; 5’-CCATGAGTTGGTAGACTTTCAAAATCTCATAGATTTGCTCTACTG-3’ [reverse]) for the pcDNA3-Casp8- I298V plasmid. Mutations were confirmed by sequencing (KI gene analysis facility, Karolinska Institutet). Two clones (denoted a and b), for each caspase-8 variant, were used throughout the experiments. The pCAX-FLAG-APP plasmid encoding the 695 amino acid amino-terminus FLAG-tagged version of amyloid precursor protein [Homo sapiens (human)] was obtained from the Addgene plasmid repository (#30154) [28].

### Cell culture, transfection and treatment

**Fig 1:**
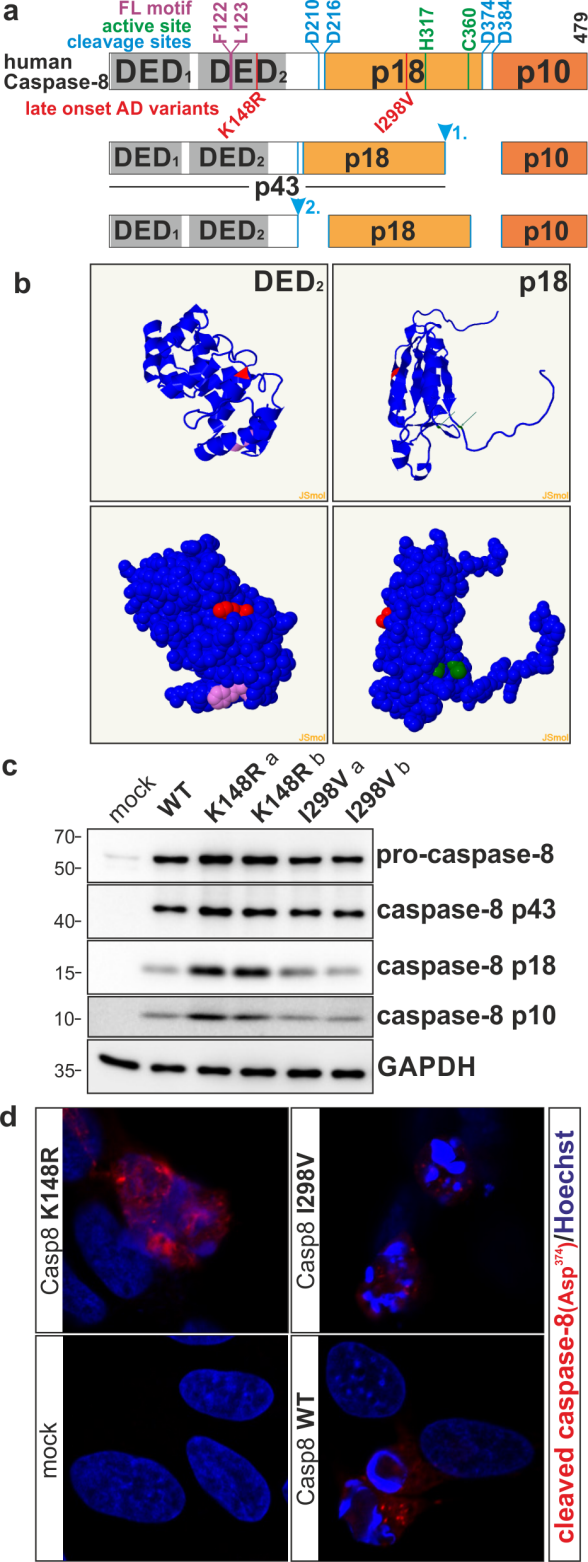
Caspase-8 modeling and expression. (**A**) Schematic illustration of pro-caspase-8 protein and its p43, p18 and p10 fragments resulting from proteolytic processing and activation. (**B**) Protein folding (top) and 3D model (bottom) of caspase-8 DED_2_ (left side) and p18 domain (right side). The K^148^ and I^298^ variants are depicted in red color. F^122^ and L^123^ of the hydrophobic FL motif within the DED_2_ is shown in purple, and critical H^317^ and C^360^ active site residues within the p18 domain are in green. (**C**) SK-N-BE(2) cells were transfected with expression vectors encoding WT-, K148R-, or I298V-caspase-8 and mock as control. Corresponding immunoblot analysis, 24 h post-transfection, indicating the expression levels for pro-caspase-8 and its p43, p18 and p10 fragments. For the LOAD caspase-8 variant, two clones (*a* and *b*) are presented.(**D**) Representative confocal images of SK-N-BE(2) cells transfected as described in panel C. The cleaved caspase-8 is labeled red and Hoechst counterstained nuclei are blue. Images were taken 24 h after transfection.

**Fig 2:**
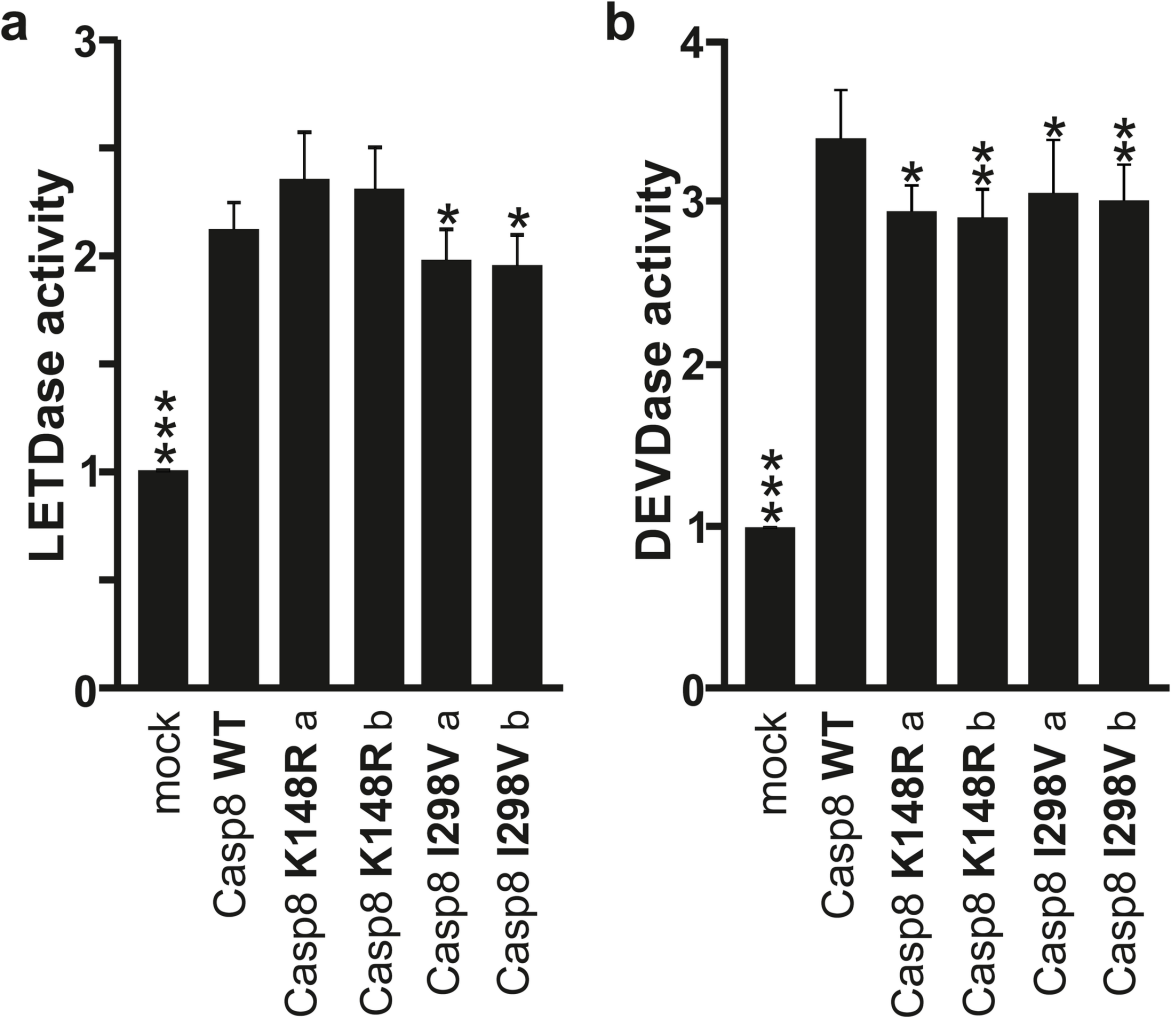
Caspase-8 enzymatic activity. SK-N-BE(2) cells were transfected with expression vectors encoding WT-, K148R-, or I298V-caspase-8 and mock as control. (A) Caspase-8 (LETDase) and (B) Caspase-3-like (DEVDase) activities were measured 24 h post-transfection. Data are presented as fold over mock untreated. Statistics and error bars: mean±s.d. n = 8 of biological replicates. Data was analyzed as comparison to Caspase-8 WT using two-sided student’s t-test. *P< 0.05; **P< 0.01 and ***P< 0.001.

Caspase-8-defective human neuroblastoma SK-N-BE(2) cells (ATCC® CRL-2271) are available at ATCC (CRL-2271) and were obtained from Marie A. Henriksson (Karolinska Institutet) and tested negative for mycoplasma contamination. Cells were maintained at 37°C, 5% CO₂, in DMEM/F12 medium (Gibco BRL) supplemented with 10% heat-inactivated fetal bovine serum, 100U/ml penicillin and 100μg/ml streptomycin. Cells were seeded at 75,000 cells/well in 12 well plates 24 h prior to transfection. 2μl Lipofectamine 2000 were used together with 0.5μg (**Figs 1 and 2**, **S1 Fig**) or 0,5–1,5μg (see supplementary materials, **S1 File**) plasmid for transfection according to manufacturer´s descriptions. Cells were transfected with plasmid(s) encoding Casp8 WT, Casp8-K148R (a or b clones), or Casp8-I298V (a or b clones), with or without the FLAG-APP. Empty vector pcDNA3.1 was used as control. Twenty-four hours after transfection cells were treated with 0.1 μM staurosporine (STS) or 200ng/ml tumor necrosis factor (TNF).

### Immunofluorescence

Cells grown on coverslips were fixed using 4% paraformaldehyde for 15 min, and blocked/permeabilized for 1 hour at room temperature in PBS-T with 10 mM HEPES, 0.3% Triton X-100, and 3% BSA. Thereafter, cells were incubated overnight at 4°C with primary cleaved Caspase-8 antibody (#9496, Cell Signaling) in the same buffer, followed by 1h incubation in RT with Alexa 594 conjugated secondary antibody (Invitrogen). Hoechst 33342 (2 μg/ml, Invitrogen) was used as a nuclear counterstain (10 min incubation). Samples were mounted onto glass slides and analyzed under Zeiss LSM700 confocal laser scanning microscopy equipped with ZEN Zeiss software.

### Immunoblotting

Total protein extracts were made directly in Laemmli loading buffer, sonicated and boiled before resolved on 8%, 12% or 15% sodium dodecyl sulfate-polyacrylamide gels and blotted onto 0.2 μm or 0.45 μm nitrocellulose membranes (Bio-Rad). Membranes were blocked in 5% milk for 1h at room temperature before incubation with primary antibodies directed against Caspase-8 (#4790, Cell Signaling), cleaved Caspase-8 (#9496, Cell Signaling), cleaved Caspase-3 (#9664, Cell Signaling), cleaved PARP (#9544, Cell Signaling), FLAG (#F3165, Sigma), APP (3E9) (#ADI-NBA-100-E, ENZO), APP ΔC31 (#ENZ-ABS445-0100, ENZO) or GAPDH (#2275PC-100, Trevigen) overnight at 4°C. Incubation with appropriate horseradish-peroxidase conjugated secondary antibodies (Pierce) was done in 2.5% milk for 1 h at room temperature and protein visualization was done using enhanced chemiluminesence (ECL, Pierce) by digital Image Quant LAS 4000 (GE healthcare).

### Caspase activity measurement

Caspase activities were measured using the Caspase-Glo®8 (#G8201) and Caspase-Glo®3/7 (#G8093) assays from Promega, following the manufacturer’s instructions. Equal volumes of cell suspension, and Caspase-Glo® reagent were placed in 96-well plates and incubated for 45 min at room temperature before luminescence was monitored using a Perkin Elmer Wallac microplate reader. Cell numbers were calculated at time of harvest and used to normalize the assay results. Figures were prepared using CorelDRAW X6.

### Ethical approval

All procedures performed in studies involving human participants were in accordance with the ethical standards of the institutional and/or national research committee and with the 1964 Helsinki declaration and its later amendments or comparable ethical standards.

This article does not contain any studies with animals performed by any of the authors.

### Informed consent

Informed consent was obtained from all individual participants included in the study.

## Results

### Targeted sequencing

The coding regions of the selected genes were sequenced in a series of 2026 Caucasian cases and 1786 Caucasian controls from 6 cohorts. After removing samples that could not be genotyped for ≥ 95% of the variants and variants that could not be genotyped in ≥ 95% of the remaining samples, a total of 1886 AD cases and 1700 controls remained for variant-burden analysis. The demographic information including age at onset for cases, age at last evaluation for controls, APOE 3/4 and 4/4 genotype for subjects that remained in analysis are presented in **Table 1**. The mean age of AD onset for cases was 70.75±9.0 years (**Table 1**). Mean age of controls was 77.94±8.48 years. The sequencing resulted in ≥20x coverage of 66–100% coding base pairs for the evaluated genes **(Table 2)**.

**Table 1:** Age and ApoE4 genotypes for subjects in discovery sample.

			Age ± SD		% ApoE3/E4		% ApoE4/E4	
Cohort	CA	CO	CA	CO	CA	CO	CA	CO
NIA-LOAD	685	384	71.9±7.83	75.8±8.22	48.18	20.05	23.80	1.30
NCRAD	333		69.12±8.9		54.35		23.12	
NACC	297	329	70.41± 9.85	77.57±5.65	43.10	23.10	16.84	2.74
NIMH	354	383	71.72±8.42	70.64±7	51.13	20.89	16.67	1.31
ACT		469		86.12±2.94		19.19		0.21
WashU	217	135	72.55±10.16	77.21±8.34	41.94	22.22	11.06	3.70
**All cohorts**	**1886**	**1700**	**70.75±9.0**	**77.94±8.48**	**47.74**	**21.09**	**18.30**	**1.85**

CA, cases; CO, controls; SD, Standard deviation. Age describes onset for cases and last evaluation or when samples were collected for controls

**Table 2:** Coverage, aggregated variant count and variant-burden test of association in discovery sample.

Gene	Coverage %^a^				
	CA	CO	Var/CA	Var/CO	p^b^
APP	98	98	18/1886	19/1700	0.74
PSEN1	91	94	23/1886	6/1700	0.0027**
PSEN2	94	92	44/1886	46/1700	0.79
TREM2	100	100	114/1886	52/1700	1.2x10^-5^**
APH1A	90	90	1/1886	1/1700	0.78
APH1B	84	84	17/1886	6/1700	0.031*
BACE1	100	100	20/1886	15/1700	0.36
CASP3	69	73	4/1886	3/1700	0.56
CASP8	100	100	26/1886	4/1700	8.6x10^-5^**
GSK3A	78	78	5/1886	4/1700	0.56
GSK3B	74	82	4/1886	3/1700	0.56
NCSTN	98	98	34/1886	32/1700	0.62
PSENEN	98	98	1/1886	1/1700	0.78

^a^ Coverage: Percentage of coding nucleotide sites that were included in analysis for each gene

^b^ p-value was determined by one-sided Fisher exact test, significant p-Values are marked in bold

Var/CA and Var/CO, Number of aggregated rare disrupting and missense variants in cases/controls and total number of subjects

* nominally statistical significance

** Significance after Bonferroni correction

### Discovery sample case-control association

The variant-burden test reached nominally significant association for *PSEN1*, *TREM2*, *APH1B* and *CASP8* (**Table 2**). Among genes known to cause AD, we did not detect an association for *APP* and *PSEN2*. Variant-burden analysis of *PSEN1* showed strong association in our dataset; we observed 23/1886 rare variants in cases and 6/1700 in controls (burden test p = 0.0027, OR = 3.49, CI = 1.42–8.58). Most of the detected *PSEN1* variants were rare and observed only once or twice. For *TREM2*, we observed 114/1886 rare variants in cases and 52/1700 in controls (burden test p = 1.2x10^-5^, OR = 2.04, CI = 1.46–2.85). This strong association is driven by three variants that are seen multiple times and are individually associated with AD in our sample rs75932628 (p.R47H) 44/1886 cases, 16/1700 controls (p = 9x10^-4^, OR = 2.51, CI = 1.41–4.47), rs143332484 (p.R62H) 49/1886 cases, 27/1700 controls (p = 0.026, OR = 1.65, CI = 1.03–2.66) and (rs2234255) (p.H157T) with 7/1883 cases and 0/1697 controls (p = 0.011, OR = n/a, CI = n/a).

Among the evaluated candidate genes we found evidence for association with *APH1B* and *CASP8*. For *APH1B*, 17/1886 rare variants in cases and 6/1700 in controls were observed (p = 0.031, OR = 2.57, CI = 1.01–6.53). However, this association failed the threshold for significance when corrected for multiple testing (multiple correction threshold for 9 candidate genes, p = 0.0055). In addition, none of the rare *APH1B* variants was observed more than 3 times.

For *CASP8*, a significant association with AD was observed after correction for multiple testing. In variant-burden analysis, we observed 26/1886 variants in cases and 4/1700 in controls (p = 8.6x10^-5^, OR = 5.93, CI = 2.06–17.02; Bonferroni correction for 9 genes p = 8x10^-4^). One variant rs146286958 (p.I298V) was observed in 10/1860 cases and 0/1693 controls, reaching significance for single variant association (p = 0.0015, OR = n/a, CI = n/a). Without I298V, the variant burden association remains significant (16/1886 cases; 4/1700 controls; p = 0.011, OR = 3.63, CI = 1.21–10.87). All *CASP8* variants identified in cases and controls were confirmed with capillary sequencing. The subjects with *CASP8* variants did not carry rare or pathogenic variants in *PSEN1*, *PSEN2*, *APP* or *TREM2* genes. All variants that were identified and included in the variant burden analysis are presented in supplementary materials, **S1 File**.

### Replication analysis

For two *CASP8* variants rs148697064 (p.K148R) and rs146286958 (p.I298V), which were observed multiple times in our sample, we performed replication study to further evaluate evidence for association. In a German sample, p.K148R was seen in 0/1143 cases and 1/850 controls and p.I298V was seen in 4/1151 cases and 4/851 controls. In a Finnish sample of 384 cases and 384 controls, all subjects were monomorphic for both variants. In the ADGC dataset p.K148R was found in 18/8779 cases and 5/7041 controls (p = 0.021, OR = 2.89, CI = 1.07–7.79) and p.I298V in 24/8782 cases and 11/7041 controls (p = 0.081, OR = 1.75, CI = 0.86–3.58).

We used our discovery sample and all 3 replication samples to perform the combined analysis. Some of the cohorts from the discovery sample were included in the ADGC dataset as well. Thus, for combined analysis we restricted the discovery samples to individuals not present in the ADGC sample. The combined analysis detected K148R in 19/10823 cases and 6/8882 controls (p = 0.025, OR = 2.6, CI = 1.04–6.57) and I298V in 33/10845 cases and 15/8881 controls (p = 0.037, OR = 1.8, CI = 0.98–3.32) (**Table 3**).

**Table 3:** Variant count and association of *CASP8* K148R and I298V.

Variant	c.443A>G, p.K148R			
	CA	CO	p	OR(95% CI)
Discovery sample	3/1851	0/1631	0.15	-
Discovery unique^a^	1/517	0/607	0.46	-
German & Finish	0/1527	1/1234	1	-
ADGC	18/8779	5/7041	0.021*	2.89(1.07–7.79)
Combined^b^	19/10823	6/8882	0.025*	2.6(1.04–6.57)
	c.892A>G, p.I298V			
	CA	CO	P	OR(95% CI)
Discovery sample	10/1860	0/1693	0.0015*	-
Discovery unique^a^	5/528	0/605	0.022*	-
German & Finish	4/1535	4/1235	0.75	0.8(0.2–3.22)
ADGC	24/8782	11/7041	0.081	1.75(0.86–3.58)
Combined^b^	33/10845	15/8881	0.037*	1.8(0.98–3.32)

^a^ Discovery unique excludes subjects that are in cohorts represented in the ADGC sample

^b^ Combined sample includes Discovery unique, German, Finnish and ADGC subjects

* nominally statistical significance

Numbering according to GenBank Accession No. NM_033355.3 for K148R and NM_033355.3

### Bioinformatics structural analysis

The 479 aa protein procaspase-8 isoform 1, including its domains, active sites and mutations that were studied for function, is graphically presented in **Fig 1A**. Model structures of caspase-8 including DED_2_ and p18 domains were generated using Phyre2, a *web*-based tool for predicting and analyzing protein structure and function [26]. These domain models revealed that caspase-8 residues Lys^148^ and Ile^298^ are both localized in an alpha helix secondary structure. Three-dimensional modeling also showed that these two residues are exposed on the surface and accessible.(**Fig 1B**).

### Expression analysis and functional studies

To investigate functional effects, p.K148R and p.I298V point mutations were inserted in an expression vector encoding the 479 aa caspase-8 isoform 1, tagged with a flag epitope and transfected into caspase-8-defective human neuroblastoma SK-N-BE(2) cells.

The cellular expression and processing ability of p.K148R and p.I298V caspase-8 was investigated by immunoblotting. Autoproteolytic processing of procaspase-8 generates caspase-8 fragments, including p43, p18 and p10. While mock transfected SK-N-BE(2) cells were nearly devoid of pro-caspase-8, robust expression of the pro-form was seen in WT, p.K148R and p.I298V transfected cells. Processing of procaspase-8 into the p43, p18 and p10 fragments could be detected in all caspase-8 transfected cells. Increased levels of protein fragments were observed for p.K148R as compared to WT and p.I298V (**Fig 1C**).

Protein expression and localization of transfected caspase-8 were further evaluated with immunofluorescence. Whereas mock-transfected cells did not exhibit an immunofluorescence signal, a distinct signal for cleaved/active Asp^374^ caspase-8 was observed in wild-type (WT)-, p.K148R- and p.I298V-caspase-8 transfected cells. The signal intensity appeared to be stronger for p.K148R, but lower for p.I298V, as compared to caspase-8 WT (**Fig 1D**).

We evaluated caspase-8 enzymatic activity directly and indirectly through monitoring of caspase-3 activity. Caspase-3 is a predominant effector for caspase-8 and has been implicated in APP proteolysis and the generation of a neurotoxic C31 APP fragment [16,17,29]. Caspase-8 activity was monitored with Caspase-Glo®8 assay, which uses a substrate that contains a Leu-Glu-Thr-Asp sequence (*i*.*e*. LETDase activity) and caspase-3 activity was monitored with the Caspase-Glo®3/7 assay and Asp-Glu-Val-Asp substrate (*i*.*e*. DEVDase activity). The p.I298V variant exhibited significantly reduced caspase-8 enzymatic activities as compared to WT (**Fig 2A**). Furthermore, for caspase-3 we observed significantly decreased activity for both p.K148R and p.I298V variants (**Fig 2B**). We further evaluated caspase-8 and caspase-3 enzymatic activity when cells were treated with two distinct caspase-8 activators, staurosporine (STS) and tumor necrosis factor (TNF). STS and TNF treatments increased LETDase (**S1A and S1B Fig**) and DEVDase activities (**S1C and S1D Fig**) for WT, p.K148R and p.I298V caspase-8 transfected cells as expected. Of note, for the most part, on exposure to these stimuli, the difference between caspase-8 WT as compared to p.K148R and p.I298V, were not significant.

The ability of the caspase-8 p.K148R and p.I298V variants to cleave APP was investigated in SK-N-BE(2) cells that were co-transfected with an expression vector encoding a Flag-tagged version of the 695 aa APP isoform and caspase-8 variants. When APP processing was examined by immunoblotting, WT caspase-8 was found to cleave APP and potentially lead to its degradation in a dose dependent manner (**S2A Fig**). With our assay, p.K148R and p.I298V caspase-8 showed comparable ability to cleave APP as compared to WT (**S2B Fig**). To look closer at APP processing by caspase-8, an antibody against the caspase cleavage product of APP, APPΔC31, was employed. Co-transfection of cells with Flag-APP and WT, p.K148R or p.I298V caspase-8 resulted in the cleavage of APP and appearance of the APPΔC31 fragment (**S2C and S2D Fig**).

## Discussion

Our study was aimed at evaluating the contribution of rare protein disrupting and missense variants in nine candidate genes involved in the amyloid metabolism (*APH1A*, *APH1B*, *BACE1*, *CASP3*, *CASP8*, *GSK3A*, *GSK3B*, *PSENEN*, and *NCSTN*). As our assumption was that individual variants responsible for the disease are rare, we have used targeted sequencing and gene based variant-burden case-control association to assess the candidate genes. In addition we have evaluated the association of rare protein disrupting and missense variants in know AD genes (*APP*, *PSEN1*, *PSEN2* and *TREM2*) in our sample. The replication of the association of *PSEN1* and *TREM2* shows the validity of our variant-burden approach. For *PSEN1*, except for the known pathogenic A79V variant, the remainder of variants we identified is observed only once or twice. Our variant-burden approach focused had sufficient power to detect *PSEN1* association although none of the variants was individually associated with AD. In contrast to *PSEN1* the association with *TREM2* was due to three variants that were seen in multiple cases. For two of the variants that carry the bulk of the association in our sample, R47H and R62H, the association with AD is well described and replicated [30]. One additional variant, rs2234255 (H157T) shows nominal association in our sample and currently there is no conclusive evidence for its association with AD [31,32]. Interestingly, in the ExAC data set this variant is reported to be at a two magnitudes higher frequency in Latino samples (MAF = 0.032) as compared to non-Finnish Europeans (MAF = 0.0003) (23). This indicates that an association study in Latino population would have excellent power to determine if this variant has a role in AD and that H157T variant might have a significant contribution to AD in Latino population due to high population frequency.

Our variant-burden analysis identified a nominally significant association of *APH1B* and significant association of *CASP8* with AD. For *APH1B* we have not observed any variant more then 3 times. This indicates that a larger re-sequencing study would be needed to obtain statistically significant evidence for *APH1B* association with AD.

We observed a strong variant-burden association for *CASP8* with AD. To follow up on variant-burden analysis we performed replication association and functional studies on p.K148R and p.I298V variants. These two variants were selected as we have seen them multiple times in our discovery sample, are present on HumanExome chip and are suitable for functional studies. The p.K148R and p.I298V variants are present on 479 amino acid procaspase-8 isoform 1 (Uniprot identifier Q14790-1), which is predominantly expressed in cells and is available as an expression vector. Variant p.P25A (rs34210251) which was also present in multiple cases in variant burden analysis was not selected for further studies as this variant is present on the caspase-8 isoform 9, also referred as 8L (Uniprot identifier: Q14790-9). For isoform 9 a backbone expression vector is not available which makes p.P25A less practical for use in functional studies.

The association of rare variants in *CASP8* with AD has not previously been reported. Large GWAS meta-analyses of 74,046 individuals has been successful in identification of genome-wide significant association of more then 20 loci with AD [33] and GWAS studies have contributed to understanding the mechanisms of AD [34]. Although GWAS studies are originally designed to detect common variants, use of genotype imputation allows for increase in resolution and detection of association of rare alleles, as demonstrated by detection of suggestive association (1x10^-6^ < p < 1x10^-8^) of rs9381040, which is 24 kb from TREM2 and AD [33]. However, ongoing systematic meta-analyses of Alzheimer disease genetic association studies in AlzGene database [35], and recent high-resolution exome variant microarray GWAS [36] have not identified association of chromosome 2q33.1 region which contains *CASP8* with AD, indicating that it is unlikely that our results are a consequence of LD with another locus in the region.

Caspase-8 belongs to a family of cysteine aspartate-specific proteases, which play essential roles in apoptosis, inflammation, and cellular differentiation. Caspase-8 is synthesized within the cell as an inactive zymogen, procaspase-8, and requires proteolytic activation. Procaspase-8 consists of a long prodomain harboring two critical protein interaction domains, DED_1_ and DED_2_, followed by one large (p18) and one small (p10) catalytic subunit. Our structural analysis shows that the K148R and the I298V caspase-8 variants affect exposed amino acid residues in the DED_2_ prodomain and p18 catalytic subunit of the enzyme, respectively. I298V is not far from the caspase active site, which includes the critical histidine H317 and cysteine C360 residues. Functional analysis revealed that the *CASP8* I298V variant is associated with a significant reduction in caspase-8 activity, as demonstrated by reduced LETDase activity, indicating that the functional effect of *CASP8* I298V may be due to a direct effect on its enzymatic activity. In contrast, K148R did not appear to affect caspase-8 enzymatic activity. Immunofluorescence analysis revealed that the K148R variant causes accumulation of cleaved caspase-8 in aggregate-like structures. Collectively, these data suggest that the *CASP8* K148R variant might affect the localization and/or turnover of caspase-8. Interestingly, the DED_2_ of procaspase-8 contains a F^122^L^123^ hydrophobic motif, in proximity to the K148R residue, and it has been proposed that FL motif is implicated in the recruitment of multiple procaspase-8 molecules as chains [37,38]. Consequently, one could envisage reduced functionality of caspase-8 K148R due to its sequestration. Reduced functionality of both mutant proteins is supported by our observation of reduced activation of caspase-3, a substrate and downstream effector of caspase-8.

There are several mechanisms that might lead to AD phenotype due to the moderate loss of caspase-8 function we have described. One such mechanism is caspase-8 effect on the function of caspase-3. Caspase-3 has been implicated in AD through involvement in APP proteolysis and the generation of a neurotoxic C31 APP related fragment [16,17,29]. However, we were not able to demonstrate statistically significant changes in APP processing, possibly due to insufficient sensitivity of our experimental model. Beside effects on APP proteolysis, caspases have been implicated in neurodegeneration and AD due to their central role in apoptosis and importance in non-apoptotic processes [39–42]. The *caspase-8* was reported to mediate Aβ-induced neuronal apoptosis in-vivo [43] and there is extensive evidence for involvement of *caspase 3* in AD. The caspase-3 is activated in Aβ-treated neuronal cultures [44], the increased levels of caspase-3 expression [45] and activated caspase-3 have been observed in AD brains [46].

The non-apoptotic caspases functions, which could lead to neurodegeneration, include effects on neuronal plasticity and structural remodeling such as axon pruning and synapse elimination, and non-neuronal functions such as role in the microglia activation [40–42]. Normal brain functions depend on proper synaptic activity and synaptic loss is one of the best pathological correlate of the cognitive decline in AD. Several studies have implicated caspases in the regulation of synaptic plasticity [47,48] thus establishing a non-apoptotic disease mechanism. In similar way, the enhancement of baseline non-apoptotic caspase-3 functions was associated with the early synaptic dysfunction in a mouse model of AD at the onset of memory decline [49].

The role for caspases in control of microglial cell activation has also been identified and related to neurodegenerative diseases including AD. Microglia act as ‘housekeepers’ in the CNS by constantly scavenging for damaged neurons, plaques and infectious agents. Thereby changes in microglial function might be detrimental for the brain homeostasis. Our group has reported that the orderly activation of caspase-8 and caspase-3 regulates microglia activation through a protein kinase C (PKC)-δ-dependent pathway [50]. We found that exacerbation of this caspase-signaling pathway was associated with neurotoxicity and that caspase-8 and caspase-3 were activated in microglia in the frontal cortex of individuals with AD, providing further mechanistic evidence for the involvement of caspases in AD. In addition to effects through caspase-3, caspase-8 cleaves additional proteins that might have a role in AD. Caspase-8 cleaves BID (BH3-Interacting Domain Death Agonist OMIM #601997) a protein whose effects mediate cytochrome-c release resulting in mitochondrial damage [51], is involved in the TNF signaling pathway [52] and it affects autophagic cell death [53]. This opens a possibility that mitochondrial metabolism, TNF signaling and autophagic cell death might be the mechanisms that are involved in *CASP8* effects in AD [54–56].

In summary, using targeted sequencing and variant-burden test we identified an association of *CASP8* with AD. For two variants, p.I298V and p.K148R the association remained significant in a large combined sample. With functional studies we showed that p.I298V and p.K148R variants have effects on caspase-8 and caspase-3 activity and thus can affect, directly or indirectly, multiple cellular processes that are regulated through caspases. In the context of majority of literature postulating an increase in caspase activity as a mechanism in AD and neurodegeneration [43–46,49,57–62], the results of our functional studies are unexpected. The variants we have identified confer a significant, although moderate, loss of *CASP8* function. This suggests that for carriers of *CASP8* variants, moderate loss of function over the course of lifespan leads to late-onset AD. Notably, a hypothesis for mechanism by which moderate inhibition of caspase activity may have profound implications in age related disorders in vivo has been proposed [63,64]. Under such hypothesis, in slow-developing neurodegenerative diseases, neuronal damage loss is preceded by non-lethal neuronal injury resulting in decreased connectivity and function. Once a cell-damage threshold is surpassed, apoptotic process and caspase activation quickly remove the damaged neurons, which in turn facilitates reorganization of surrounding neurons and/or replacement by neurogenesis. Inhibition of apoptosis by local mediators, energy loss or caspase inhibition could delay removal of severely damaged cells and interfere with reorganization of surrounding neurons and/or replacement by neurogenesis. The loss of function variants we have identified here could be one such mechanism that leads to decreased caspase activation. In *CASP8* loss of function variant carriers, the inhibition of apoptosis and other caspase mediated functions such as axon pruning and synapse elimination could lead to AD pathogenesis by decreased removal of damaged cells. Such damaged neurons could affect brain function due to decreased connectivity and cell function. Furthermore, as caspases are involved in microglial function [40–42], the disruption in microglial activation may result in sustained inflammation which is deleterious to neurons, and is increasingly recognized as a feature of chronic neurodegenerative diseases including AD [65–67].

### Limitations and future directions

Our study design has several limitations. We have evaluated only a limited number of candidate genes in sequencing variant-burden association study. The amyloid cascade hypothesis, on which we based our candidate gene selection, involves complex biological processes that regulate balance between Aβ production and clearance. Despite the importance of amyloid hypothesis, the exact number and role of the genes and pathways involved is not known. The KEGG database [68], a collection of manually curated knowledge on the molecular interaction, reaction and relation networks for disease currently includes 171 genes in Alzheimer disease pathway (KEGG PATHWAY: hsa05010). The PANTHER is another classification system, which was designed to group proteins based on function or biological processes using human curating and sophisticated bioinformatics algorithms [69]. In a similar way PANTHER AD Amyloid Secretase (P00003) and AD Presenilin (P00004) pathway gene sets include 55 and 98 proteins respectively, indicating that large number of genes might be involved in amyloid cascade. To balance the number of candidate genes and number of cases and controls to include in our study, we have opted to a strategy that includes relatively small number of candidate genes (9 genes) and large number of cases and controls (3586 subjects) analyzed. In comparable fashion, Sassi et al. [70] have recently reported analysis of 29 genes they selected as relevant for amyloid hypothesis in 332 cases and 676 controls (1008 subjects). Two genes on our respective lists overlapped (BACE1 and APH1B). Sassi et al, have not reported significant association when corrected for multiple testing and subsequently have not followed their results with replication study or variant in-vitro functional analysis. Their design covered larger number of genes in smaller sample, which decreases power as compared to our design where we have identified a statistically significant association with CASP8.

Additional weakness of our study is a lack of true replication. Due to cost of sequencing we have replicated two *CASP8* variants that were present on exome chip. For both variants, in replication sample we have found excess of heterozygous carriers in cases as compared to controls, although this excess was not statistically significant. In order to obtain a true replication a larger re-sequencing study of CASP8 is needed.

To further the understanding of the mechanisms of human disease, animal models have been invaluable. Several dozens of AD mouse models have been developed based on human AD mutations in APP, presenilins, and/or tau protein [71–73]. Although transgenic AD models developed show Aβ accumulation, gliosis, neuronal loss, tau pathology, and/or cognitive impairments, no single transgenic AD model recapitulates all aspects of AD pathology. Furthermore as AD is a late onset disease, to show AD pathology and phenotype in mice models like 5xFAD and 3xTg include multiple pathogenic mutations in APP, PSEN1 and MAPT genes.

For in*-vivo* validation of our findings and to understand the disease mechanisms, the generation of *Casp8* mouse models would be invaluable. Recent discovery of TREM2 as AD susceptibility gene shows the complexities and path toward development of such models [74]. To tackle the role of TREM2 in context of AD the investigators have examined TREM2 deficient mice that also carry mutations in human APP and PSEN1 such as 5xFAD and APPPS1 mice [75–78]. Interestingly, such studies have produced broad spectrum of results, with both increased and decreased amyloid pathology, possibly depending on the stage of disease progression. For Casp8 a knockout mouse model has been developed as well, showing no apparent phenotype for heterozygous mice and embryonic lethality for homozygous mutants [79]. As a next step in biological validation of association of *CASP8* and AD one could envision studies of Casp8 haploinsufficiency in transgenic AD mouse. Such studies could more precisely examine AD mechanisms that could lead to AD such as, apoptotic changes, Aβ accumulation, axon pruning and synaptic elimination or changes in microglia function [39–42].

Although the role of caspases in AD has been proposed, our study for the first time shows genetic association of rare variants in *CASP8* with AD and proposes a mechanism of action mediated by decreased enzyme activity. Furthermore, we have shown that the enzymatic activity of AD associated caspase-8 variants K148R and I298V increases when exposed to activators such as STS and TNF. One could propose that in theory some caspase activators with high specificity and low toxicity (*i*.*e*. which would not promote inappropriate cell death) could increase and "normalize" caspase activity in individuals carrying variants that impair caspase function and possibly be used in AD prevention and treatment. Our finding is even more interesting as caspase inhibition, as opposed to activation has been proposed as a mechanism for AD treatment [80,81]. This indicates that further studies in investigating the role of caspases in neurological disease might be needed before caspase activity modulation is considered for AD treatment.

## Supporting information

S1 File Supplemental methods. (PDF)

S2 File Identified and counted variants. (ZIP)

S3 File Supplemental acknowledgments. (PDF)

S1 Fig Late onset AD caspase-8 variants affect caspase responses upon death stimuli. (**a** to **d**) SK-N-BE(2) cells were transfected with expression vector encoding Caspase-8 WT, Caspase-8 K148R, or Caspase-8 I298V and mock as control. Twenty-four hours post-transfection, cells were treated with 0.1μM staurosporine (STS) (**a**, **b**) or 200ng/ml tumor necrosis factor (TNF) (**c, d**) for an additional 6 hours. Thereafter, caspase-8-related LETDase activity (a, c), and downstream Caspase-3-like-related DEVDase activity (**b, d**) were monitored.
Data are presented as fold over mock untreated. Statistics and error bars: mean±s.d. n = 3–5 of biological replicates. Data was analyzed as comparison to Caspase-8 WT using two-sided student’s t-test. *P< 0.05; **P< 0.01 and ***P< 0.001.
(PDF)

S2 Fig Effect of late onset AD caspase-8 variants on the cleavage of amyloid precursor protein. (**a** to **d**) SK-N-BE(2) cells were co-transfected with expression vector encoding Flag tagged APP together plasmid for Caspase-8 WT, Caspase-8 K148R, or Caspase-8 I298V and mock as control. Caspase-8 WT expression lead to dose-dependent processing of Flag-APP as detected with antibodies directed against the Flag-tag epitope (**a**) or the APP protein itself (**c**). The Caspase-8 K148R and Caspase-8 I298V mutants are also able to cleave APP, as indicated by the reduced levels of APP detected with anti-Flag (**b**) or anti-APP antibodies (**d**). Introduction of Caspase-8 WT leads to cleavage of APP at its VEVD664 caspase-cleavage site, resulting in the formation of an APP ΔC31 fragment (**c**). Elevated levels of APP ΔC31 can be detected in Caspase-8 WT as well as the Caspase-8 K148R and Caspase-8 I298V transfected cells (**d**). *indicates APP and cleaved APP detected by Flag antibody.
(PDF)
