# Supplementary material for: Caspase-8, association with Alzheimer’s Disease and functional analysis of rare variants
Source: PLoS One. 2017 Oct 6;12(10):e0185777. doi: 10.1371/journal.pone.0185777 (PMC5630132; doi:10.1371/journal.pone.0185777)
Supplement: S1 File — (PDF) [file pone.0185777.s001.pdf]

S1 | Supplemental methods

Study samples

Discovery samples:

NIA-LOAD and NCRAD samples

Subjects were obtained from NCRAD, and ascertainment and collection details can be found at the NCRAD website (http://www.ncrad.org). For our study, we used subjects of self-reported European ancestry with DNA selected from one affected individual from each family. Affected individuals were selected based on strength of diagnosis (confirmed and probable) and DNA availability. For both cohorts (NIA-LOAD or NCRAD), cases and families were required to meet the same diagnostic study criteria. In the families, probands were required to have a diagnosis of definite or probable LOAD with onset > 60 years of age. Both studies also required additional family members with definite, probable or possible AD. The NIA-LOAD study also includes unrelated controls without family history of AD that were required to have cognitive testing and clinical examination to establish normal cognitive functioning for age and absence of AD.

NACC samples

The NACC was established by the National Institute on Aging to facilitate collaborative research. Samples were collected from 27 NIA-funded Alzheimer's Disease Centers (ADCs) across the United States. Cases and controls describing themselves as “white” that have met stringent gold-standard diagnostic criteria as described before [1–3] were selected for targeted sequencing. The NACC database September 2013 freeze was used to select cases and controls. The DNA samples were obtained from the National Cell Repository for Alzheimer's Disease (NCRAD).

NIMH cases

NIMH families have been obtained by three sites (University of Alabama - Birmingham, Johns Hopkins University, and Massachusetts General Hospital/Harvard Medical School). Data collection has been coordinated among the three sites by using a common protocol that includes uniform assessments and medical, neurologic, and psychiatric histories. Operational criteria for the clinical diagnosis of probable or possible AD following NINCDS-ADRDA Work Group guidelines have been implemented by all three sites. Each family had at least two affected individuals. Definite AD according to age-adjusted Khachaturian criteria was established on autopsy [4,5]. From each family one subject was selected for sequencing.

NIMH controls

In order to enable case-control association studies the NIMH has established a set of controls. This control sample consists of a group of adults who completed an online, short self-report clinical assessment. For our study we included Caucasian individuals older than 60 years. NIMH controls did not have cognitive testing [6].

ACT controls

The Adult Changes in Thought (ACT) study enrolled cognitively intact, age 65 and older, members of the Group Health Cooperative (GHC) health care system in the Seattle, Washington, USA [7]. All of the subjects included in our study were cognitively normal at the age of the last exam.

Washington University

The samples were obtained at the Knight-ADRC at Washington University. Cases and controls were evaluated in the Clinical Core of the Knight ADRC. Cases received a clinical diagnosis of AD dementia in accordance with standard criteria [3] and dementia severity was determined with the Clinical Dementia Rating (CDR) [8]. All individuals were of European descent.

In all cohorts written consent was obtained from all participants at participating institutions.

Replication study:

German Sample

The German AD cases for genotyping were recruited from the following three sources: (i) the German Dementia Competence Network [9] (DCN, n=311); (ii) the German study on Aging, Cognition, and Dementia in primary care patients [10] (AgeCoDe, n=148); and (iii) the interdisciplinary Memory Clinic at the University Hospital of Bonn (n=692). The German controls were drawn from the AgeCoDe cohort (n=851). (i) The DCN cases were evaluated using extensive neuropsychological tests including those of the Consortium to Establish a Registry for Alzheimer's Disease (CERAD); the Mini Mental State Exam (MMSE); and the Clinical Dementia rating (CDR-SB) [9]. Whole blood, genomic DNA, plasma, and CSF were collected from all participants. (ii) AgeCoDe is a German multicenter prospective cohort study. The assessment includes the Structured Interview for Diagnosis of Dementia of Alzheimer type, Multi-infarct Dementia, and Dementia of other etiology according to DSM-IV and ICD-10 (SIDAM) [11]. Dementia was diagnosed according to DSM-IV criteria and for AgeCoDe controls included in the present study, dementia and mild cognitive impairment had been excluded at the last follow-up visit. (iii) In interdisciplinary Memory Clinic of the Department of Psychiatry and Department of Neurology at the University Hospital in Bonn the diagnoses were assigned according the NINCDS/ADRDA criteria [12] and on the basis of clinical history, physical examination, neuropsychological testing (using the CERAD neuropsychological battery, including the MMSE), laboratory assessments, and brain imaging.

Finnish Sample

The Finnish-AD cohort comprises of 672 AD patients and 686 age-matched healthy controls from Kuopio in Finland. All patients were diagnosed with probable AD according to the National Institute of Neurological and Communicative Disorders and Stroke and the Alzheimer’s Disease and Related

Disorders Association (NINCDS-ADRDA) criteria [13]. Control subjects had no symptoms of cognitive impairment based on clinical interview and neuropsychological examination.

ADGC sample

Cases and controls were taken from multiple ADGC datasets [2,14], described here briefly. The *NACC* National Institute on Aging sample, the University of Toronto/GlaxoSmithKline case-control sample, the Vanderbilt/ Miami/Mount Sinai case-control sample, the National Institute on Aging LOAD sample, the NCRAD multiplex family-based sample, the Multi-institutional Research in Alzheimer's Genetic Epidemiology family-based sample, and ACT prospective cohort were described in references [15,16]. The Genetics Differences cohort is a population-based prevalent case-control study from the same population as the ACT study [17]. Additional studies included in ADGC sample are; The Washington Heights–Inwood Columbia Aging Project, Northern European ancestry subjects [18]; the Washington University cohort is a case-control cohort of individuals with Northern European ancestry [14]; The University of Miami case-control dataset [19]; and the Cache County Study on Memory Health and Aging [20]. Per individual source studies, all subjects were recruited under protocols approved by the appropriate Institutional Review Boards. Cases living at time of recruitment were adjudicated as possible or probable AD prior to analyses according to NINCDS/ADRDA criteria [13] whereas affection status of all deceased cases was confirmed through autopsy.

MIP design, sequencing and alignment

MIPs were designed with the MIP generator pipeline [21]. The targeted sequence for each MIP was 112 bp long with 20 bp extension and ligation arms. Each MIP included a 5 bp long sequence tag as an identification barcode to separate up to 1024 unique reads from PCR duplicates. Captured sequences were PCR amplified with primers containing an 8 bp barcode to distinguish individual samples. MIPs were at first combined in equimolar amounts. Target capture and sequencing was performed as described [22]. A subset of samples were sequenced on Illumina MiSeq® to allow recalibration of probes depending upon probe performance. The recalibrated MIPs were divided in two pools based

on performance. Capturing and barcoding was performed for each sample in both pools and barcoded were merged with in 1:3 ratio.

192 samples, divided between cases and controls were sequenced one lane of an Illumina Hiseq® 2500 sequencer. Resulting reads were checked for quality with FastQC [23]. The split_libraries_fastq.py script from the tool kit Qiime [24] was used to identify the barcodes in each read and after identification the reads were written back into a FASTQ file.

Reads were aligned to the human genome (hg19) with BWA [25]. The aligned file was divided into 192 unique barcoded samples and separated into FASTQ files. Prinseq [26] was used to split extracted reads into unique and duplicates followed by duplicate and MIP arm removal. Resulting FASTQ files were again aligned to hg19 and individual samples separated. Individual alignments were further refined using the GATK [27] 'IndelRealigner' and 'BaseRecalibrator' tools.

Variant calling and genotyping

Next generation sequencing coverage was determined with the GATK DepthOfCoverage tool**.** Variants were called and filtered (QualByDepth < 1.5, HomopolymerRun > 6 and Total Depth over all samples ≥ 20) with the Genome Analysis Toolkit (GATK) [28]. Individuals that could not be genotyped for ≥ 95% of the variants and variants that could not be found in ≥ 95% of the remaining individuals were removed before analysis. To exclude possible sequencing artifacts, we followed the GATK best practices workflow for variant discovery. Variants that passed QC were annotated for frequency and function with the Seattleseq 137 annotation pipeline [28]. Variant frequency was assigned based on 1000 Genome European dataset (phase1_release_v3.20101123). All annotated variants had at least 8 reads and at least 25% of reads (minimum two) had to show the alternate allele to be called heterozygous.

In the Finish and German samples genotyping was performed using Sequenom iPlexa and TaqMan® SNP genotyping assay respectively. The variants in the CASP8 and APH1B genes in cases and controls were confirmed with capillary sequencing in the primary study and in the German replication sample.

For ADGC samples 18,099 samples were genotyped on exome arrays, including 16,571 samples on the Infinium HumanExome V1 Beadchip (Illumina, Inc) and 1,528 samples on the V3 Beadchip. Genotyping was performed for 8,410 samples at the Robert S. Boas Center for Genomics and Human Genetics, Feinstein Institute for Medical Research, Manhasset, New York; 1,990 individuals at the John P. Hussman Institute for Human Genomics, University of Miami, Miami, Florida; and 7,694 individuals at the Center for Applied Genomics, The Children's Hospital of Philadelphia, Philadelphia, Pennsylvania. Genotypes were initially called using the default clustering profiles from Illumina and recalled using clustering profiles generated by Genentech using data from 30,000 samples.

Exome chip genotyping data (18,099 samples total) were first pre-processed using quality check steps adapted from Naj et al. [14]. Briefly, 2,275 samples were excluded due to: (a) overall genotype call rates <98%, (b) genotype-imputed or reported sex mismatch, (c) excess relatedness ($\hat{\pi} > 0.4$ using 15,086 linkage disequilibrium–pruned autosomal markers with a minor allele frequency [MAF]>0.1), or (d) lack of Northern European ancestry. Non-Caucasian samples were identified as outliers during population substructure estimation using the program EIGENSTRAT for principal components analysis [29]. In all, 8,390 AD cases and 7,434 cognitively-normal individuals passed quality control and used in analysis in our study.

1 Beekly DL, Ramos EM, Lee WW, Deitrich WD, Jacka ME, Wu J *et al.* The National Alzheimer's Coordinating Center (NACC) database: the Uniform Data Set. *Alzheimer Dis Assoc Disord* 2007; **21**: 249–258.

2 Lambert J-C, Ibrahim-Verbaas CA, Harold D, Naj AC, Sims R, Bellenguez C *et al.* Meta-analysis of 74,046 individuals identifies 11 new susceptibility loci for Alzheimer's disease. *Nat Genet* 2013; **45**: 1452–1458.

3 Morris JC, Weintraub S, Chui HC, Cummings J, DeCarli C, Ferris S *et al.* The Uniform Data Set (UDS): Clinical and Cognitive Variables and Descriptive Data From Alzheimer Disease Centers: *Alzheimer Dis Assoc Disord* 2006; **20**: 210–216.

4 Kim M, Suh J, Romano D, Truong MH, Mullin K, Hooli B *et al.* Potential late-onset Alzheimer's disease-associated mutations in the ADAM10 gene attenuate α-secretase activity. *Hum Mol Genet* 2009; **18**: 3987–3996.

5 Schjeide B-MM, McQueen MB, Mullin K, DiVito J, Hogan MF, Parkinson M *et al.* Assessment of Alzheimer's disease case–control associations using family-based methods. *Neurogenetics* 2009; **10**: 19–25.

6 Sanders AR, Levinson DF, Duan J, Dennis JM, Li R, Kendler KS *et al.* The Internet-Based MGS2 Control Sample: Self Report of Mental Illness. *Am J Psychiatry* 2010; **167**: 854–865.

7 Kukull WA, Higdon R, Bowen JD, et al. Dementia and alzheimer disease incidence: A prospective cohort study. *Arch Neurol* 2002; **59**: 1737–1746.

8 Morris JC. The Clinical Dementia Rating (CDR): current version and scoring rules. *Neurology* 1993; **43**: 2412–2414.

9 Kornhuber J, Schmidtke K, Frolich L, Perneczky R, Wolf S, Hampel H *et al.* Early and differential diagnosis of dementia and mild cognitive impairment: design and cohort baseline characteristics of the German Dementia Competence Network. *Dement Geriatr Cogn Disord* 2009; **27**: 404–417.

10 Jessen F, Wiese B, Bickel H, Eiffländer-Gorfer S, Fuchs A, Kaduszkiewicz H *et al.* Prediction of Dementia in Primary Care Patients. *PLoS ONE* 2011; **6**: e16852.

11 Zaudig M, Mittelhammer J, Hiller W, Pauls A, Thora C, Morinigo A *et al.* SIDAM--A structured interview for the diagnosis of dementia of the Alzheimer type, multi-infarct dementia and dementias of other aetiology according to ICD-10 and DSM-III-R. *Psychol Med* 1991; **21**: 225–236.

12 McKhann G, Drachman D, Folstein M, Katzman R, Price D, Stadlan EM. Clinical diagnosis of Alzheimer's disease Report of the NINCDS-ADRDA Work Group* under the auspices of Department of Health and Human Services Task Force on Alzheimer's Disease. *Neurology* 1984; **34**: 939–939.

13 McKhann G, Drachman D, Folstein M, Katzman R, Price D, Stadlan EM. Clinical diagnosis of Alzheimer's disease: report of the NINCDS-ADRDA Work Group under the auspices of Department of Health and Human Services Task Force on Alzheimer's Disease. *Neurology* 1984; **34**: 939–944.

14 Hollingworth P, Harold D, Sims R, Gerrish A, Lambert J-C, Carrasquillo MM *et al.* Common variants at ABCA7, MS4A6A/MS4A4E, EPHA1, CD33 and CD2AP are associated with Alzheimer's disease. *Nat Genet* 2011; **43**: 429–435.

15 Jun G, Naj AC, Beecham GW, et al. MEta-analysis confirms cr1, clu, and picalm as alzheimer disease risk loci and reveals interactions with apoe genotypes. *Arch Neurol* 2010; **67**: 1473–1484.

16 Naj AC, Jun G, Beecham GW, Wang L-S, Vardarajan BN, Buros J *et al.* Common variants at MS4A4/MS4A6E, CD2AP, CD33 and EPHA1 are associated with late-onset Alzheimer's disease. *Nat Genet* 2011; **43**: 436–441.

17 Kukull WA, Schellenberg GD, Bowen JD, McCormick WC, Yu C-E, Teri L *et al.* Apolipoprotein E in Alzheimer's disease risk and case detection: A case-control study. *J Clin Epidemiol* 1996; **49**: 1143–1148.

18 Tang M, Stern Y, Marder K, et al. THe apoe-∊4 allele and the risk of alzheimer disease among african americans, whites, and hispanics. *JAMA* 1998; **279**: 751–755.

19 Naj AC, Beecham GW, Martin ER, Gallins PJ, Powell EH, Konidari I *et al.* Dementia Revealed: Novel Chromosome 6 Locus for Late-Onset Alzheimer Disease Provides Genetic Evidence for Folate-Pathway Abnormalities. *PLOS Genet* 2010; **6**: e1001130.

20 Breitner JCS, Wyse BW, Anthony JC, Welsh-Bohmer KA, Steffens DC, Norton MC *et al.* APOE-ε4 count predicts age when prevalence of AD increases, then declines The Cache County Study. *Neurology* 1999; **53**: 321–321.

21 Boyle EA, O'Roak BJ, Martin BK, Kumar A, Shendure J. MIPgen: Optimized Modeling and Design of Molecular Inversion Probes for Targeted Resequencing. *Bioinformatics* 2014; : btu353.

22 O'Roak BJ, Vives L, Fu W, Egertson JD, Stanaway IB, Phelps IG *et al.* Multiplex Targeted Sequencing Identifies Recurrently Mutated Genes in Autism Spectrum Disorders. *Science* 2012; **338**: 1619–1622.

23 Babraham Bioinformatics - FastQC A Quality Control tool for High Throughput Sequence Data. http://www.bioinformatics.bbsrc.ac.uk/projects/fastqc/ (accessed 24 Mar2016).

24 Caporaso JG, Kuczynski J, Stombaugh J, Bittinger K, Bushman FD, Costello EK *et al.* QIIME allows analysis of high-throughput community sequencing data. *Nat Methods* 2010; **7**: 335–336.

25 Li H, Durbin R. Fast and accurate long-read alignment with Burrows–Wheeler transform. *Bioinformatics* 2010; **26**: 589–595.

26 Schmieder R, Edwards R. Quality control and preprocessing of metagenomic datasets. *Bioinforma Oxf Engl* 2011; **27**: 863–864.

27 McKenna A, Hanna M, Banks E, Sivachenko A, Cibulskis K, Kernytsky A *et al.* The Genome Analysis Toolkit: A MapReduce framework for analyzing next-generation DNA sequencing data. *Genome Res* 2010; **20**: 1297–1303.

28 SeattleSeq Variation Annotation. http://snp.gs.washington.edu/SeattleSeqAnnotation137/ (accessed 24 Mar2016).

29 Price AL, Patterson NJ, Plenge RM, Weinblatt ME, Shadick NA, Reich D. Principal components analysis corrects for stratification in genome-wide association studies. *Nat Genet* 2006; **38**: 904–909.

30 Kelley LA, Mezulis S, Yates CM, Wass MN, Sternberg MJE. The Phyre2 web portal for protein modeling, prediction and analysis. *Nat Protoc* 2015; **10**: 845–858.

31 Stennicke HR, Jürgensmeier JM, Shin H, Deveraux Q, Wolf BB, Yang X *et al.* Pro-caspase-3 Is a Major Physiologic Target of Caspase-8. *J Biol Chem* 1998; **273**: 27084–27090.

32 Young-Pearse TL, Bai J, Chang R, Zheng JB, LoTurco JJ, Selkoe DJ. A Critical Function for β-Amyloid Precursor Protein in Neuronal Migration Revealed by In Utero RNA Interference. *J Neurosci* 2007; **27**: 14459–14469.
