# Supplementary material for: Caspase-8, association with Alzheimer’s Disease and functional analysis of rare variants
Source: PLoS One. 2017 Oct 6;12(10):e0185777. doi: 10.1371/journal.pone.0185777 (PMC5630132; doi:10.1371/journal.pone.0185777)
Supplement: S3 File — (PDF) [file pone.0185777.s003.pdf]

S3 | Supplemental acknowledgements

PcDNA3-Casp8 and pCAX FLAG APP plasmids were deposited to Addgene by Guy Salvesen and Dennis Selkoe & Tracy Young-Pearse, respectively. SK-N-BE(2) cells were a gift from Marie A. Henriksson.

Samples from the National Cell Repository for Alzheimer's Disease (NCRAD), which receives government support under a cooperative agreement grant (**U24 AG21886**) awarded by the National Institute on Aging (NIA), were used in this study.

NIA-LOAD

The NIA-LOAD Family study supported the collection of samples used in this study through National Institute on Aging (NIA) grants U24 AG026395, R01AG041797 and U24 AG026390.

NACC

The NACC database is funded by NIA/NIH Grant **U01 AG016976**. NACC data are contributed by the NIAfunded ADCs: P30 AG019610 (PI Eric Reiman, MD), P30 AG013846 (PI Neil Kowall, MD), P50 AG008702 (PI Scott Small, MD), P50 AG025688 (PI Allan Levey, MD, PhD), P50 AG047266 (PI Todd Golde, MD, PhD), P30 AG010133 (PI Andrew Saykin, PsyD), P50 AG005146 (PI Marilyn Albert, PhD), P50 AG005134 (PI Bradley Hyman, MD, PhD), P50 AG016574 (PI Ronald Petersen, MD, PhD), P50 AG005138 (PI Mary Sano, PhD), P30 AG008051 (PI Steven Ferris, PhD), P30 AG013854 (PI M. Marsel Mesulam, MD), P30 AG008017 (PI Jeffrey Kaye, MD), P30 AG010161 (PI David Bennett, MD), P50 AG047366 (PI Victor Henderson, MD, MS), P30 AG010129 (PI Charles DeCarli, MD), P50 AG016573 (PI Frank LaFerla, PhD), P50 AG016570 (PI Marie-Francoise Chesselet, MD, PhD), P50 AG005131 (PI Douglas Galasko, MD), P50 AG023501 (PI Bruce Miller, MD), P30 AG035982 (PI Russell Swerdlow, MD) , P30 AG028383 (PI Linda Van Eldik, PhD), P30 AG010124 (PI John Trojanowski, MD, PhD), P50 AG005133 (PI Oscar Lopez, MD), P50 AG005142 (PI Helena Chui, MD), P30 AG012300 (PI Roger Rosenberg, MD), P50 AG005136 (PI Thomas Montine, MD, PhD), P50 AG033514 (PI Sanjay Asthana, MD, FRCP), P50 AG005681 (PI John Morris, MD), and P50 AG047270 (PI Stephen Strittmatter, MD, PhD).

NIMH

Data and biomaterials for the NIMH samples were collected in three projects that participated in the National Institute of Mental Health (NIMH) Alzheimer Disease Genetics Initiative, funded from 1991 to 1998 by **U01 MH46281** (M.S. Albert and D. Blacker), **U01 MH46290** (S. Bassett, G.A. Chase, and M.F. Folstein) and **U01 MH46373** (R.C.P. Go and L.E. Harrell).

The Washington University Alzheimer's Knight-ADRC was supported by R01-NS085419; R01-AG044546, P01-AG003991 and R01-AG035083. The ACT study was supported by 2 U01 AG006781.

We would like to thank all additional members of the AgeCoDe Study Group. This publication is part of the German Research Network on Degenerative Dementia (KNDD) and was funded by the German Federal Ministry of Education and Research (grants KNDD: 01GI0710, 01GI0711, 01GI0712, 01GI0713, 01GI0714, 01GI0715, 01GI0716, 01ET1006B).

Academy of Finland, VTR grant V16001 of Kuopio University Hospital, Sigrid Jusélius Foundation, the Strategic Funding of the University of Eastern Finland, FP7, Grant Agreement no 601055, VPH Dementia Research Enabled by IT VPH-DARE@IT, and EADB project in the JPND-CO-FUND program (no 301220)

Authors/Members in the Alzheimer's Disease Genetics Consortium

Marilyn S. Albert, PhD; Roger L. Albin, MD; Liana G. Apostolova, MD; Steven E. Arnold, MD; Robert Barber, PhD; M. Michael Barmada, PhD; Lisa L. Barnes, PhD; Thomas G. Beach, MD, PhD; James T. Becker, PhD; Gary W. Beecham, PhD; Duane Beekly, BS; David A. Bennett, MD; Eileen H. Bigio, MD; Thomas D. Bird, MD; Deborah Blacker, MD, ScD; Bradley F. Boeve, MD; James D. Bowen, MD; Adam Boxer, MD, PhD; James R. Burke, MD, PhD; Joseph D. Buxbaum, PhD; Nigel J. Cairns, PhD; Chuanhai Cao, PhD; Chris S. Carlson; Steven L. Carroll, MD, PhD; Helena C. Chui, MD; David G. Clark, MD; David H. Cribbs, PhD; Elizabeth A. Crocco, MD; Charles DeCarli, MD; Steven T. DeKosky, MD; F. Yesim

Demirci, MD; Malcolm Dick, PhD; Dennis W. Dickson, MD; Ranjan Duara, MD; Nilufer Ertekin-Taner, MD, PhD; Kenneth B. Fallon, MD; Martin R. Farlow, MD; Steven Ferris, PhD; Matthew P. Frosch, MD, PhD; Douglas R. Galasko, MD; Mary Ganguli, MD, PhD; Marla Gearing, PhD; Daniel H. Geschwind, MD, PhD; Bernardino Ghetti, MD; John R. Gilbert, PhD; Jonathan D. Glass, MD; Neill R. GraffRadford, MD; John H. Growdon, MD; Ronald L. Hamilton, MD; Kara L. Hamilton-Nelson; Lindy E. Harrell, MD, PhD; Elizabeth Head, PhD; Lawrence S. Honig, MD, PhD; Christine M. Hulette, MD; Bradley T. Hyman, MD, PhD; Gail P. Jarvik, MD, PhD; Gregory A. Jicha, MD, PhD; Lee-Way Jin, MD, PhD; Gyungah Jun, PhD; M. Ilyas Kamboh, PhD; Anna Karydas, BA; Jeffrey A. Kaye, MD; Ronald Kim, MD; Edward H. Koo, MD; Neil W. Kowall, MD; Joel H. Kramer, PhD; Patricia Kramer, PhD; Frank M. LaFerla, PhD; James J. Lah, MD, PhD; James B. Leverenz, MD; Allan I. Levey, MD, PhD; Ge Li, MD, PhD; Andrew P. Lieberman, MD, PhD; Oscar L. Lopez, MD; Kathryn L. Lunetta, PhD; Constantine G. Lyketsos, MD, MHS; Wendy J. Mack, PhD; Daniel C. Marson, JD, PhD; Eden R. Martin, PhD; Frank Martiniuk, PhD; Deborah C. Mash, PhD; Eliezer Masliah, MD; Wayne C. McCormick; Susan M. McCurry, PhD; Andrew N. McDavid, BA; Ann C. McKee, MD; M. Marsel Mesulam, MD; Bruce L. Miller, MD; Carol A. Miller, MD; Joshua W. Miller, MD; Thomas J. Montine, MD, PhD; John C. Morris, MD; Jill R. Murrell, PhD; John M. Olichney, MD; Joseph E. Parisi, MD; William Perry; Elaine Peskind, MD; Ronald C. Petersen, MD, PhD; Aimee Pierce; Wayne W. Poon, PhD; Huntington Potter, PhD; Joseph F. Quinn, MD; Ashok Raj, MD; Murray Raskind, MD; Eric M. Reiman, MD; Barry Reisberg, MD; Christiane Reitz, MD, PhD; John M. Ringman, MD, MS; Erik D. Roberson, MD, PhD; Howard J. Rosen, MD; Roger N. Rosenberg, MD; Mary Sano, PhD; Andrew J. Saykin, PsyD; Julie A. Schneider, MD; Lon S. Schneider, MD; William W. Seeley, MD; Amanda G. Smith; Joshua A. Sonnen, MD; Salvatore Spina, MD; Robert A. Stern, PhD; Rudolph E. Tanzi, PhD; Tricia A. Thornton-Wells, PhD; John Q. Trojanowski, MD, PhD; Juan C. Troncoso, MD; Debby W. Tsuang, MD; Vivianna M. Van Deerlin, MD, PhD; Linda J. Van Eldik, PhD; Badri N. Vardarajan, PhD; Harry V. Vinters, MD; Jean Paul Vonsattel, MD; Sandra Weintraub, PhD; Kathleen A. Welsh-Bohmer, PhD; Jennifer Williamson, MS, MPH; Sarah Wishnek; Randall L. Woltjer, MD, PhD; Clinton B. Wright, MD, MS; Steven G. Younkin, MD, PhD; Chang-En Yu, PhD; Lei Yu, PhD.

Affiliations of Authors/Members in the Alzheimer’s Disease Genetics Consortium

Department of Neurology, Johns Hopkins University, Baltimore, Maryland (Albert); Department of Neurology, University of Michigan, Ann Arbor (Albin); Geriatric Research, Education, and Clinical Center, VA Ann Arbor Healthcare System, Ann Arbor, Michigan (Albin); Michigan Alzheimer’s Disease Center, Ann Arbor (Albin); Department of Neurology, University of California, Los Angeles (Apostolova, Ringman, Vinters); Department of Psychiatry, University of Pennsylvania Perelman School of Medicine, Philadelphia (Arnold); Department of Pharmacology and Neuroscience, University of North Texas Health Science Center, Fort Worth (Barber); Department of Human Genetics, University of Pittsburgh, Pittsburgh, Pennsylvania (Barmada, Demirci, Kamboh); Department of Neurological Sciences, Rush University Medical Center, Chicago, Illinois (Barnes, Bennett, J. A. Schneider, L. Yu); Department of Behavioral Sciences, Rush University Medical Center, Chicago, Illinois (Barnes); Civin Laboratory for Neuropathology, Banner Sun Health Research Institute, Phoenix, Arizona (Beach); Department of Psychiatry, University of Pittsburgh School of Medicine, Pittsburgh, Pennsylvania (Becker, Ganguli); Department of Neurology, University of Pittsburgh School of Medicine, Pittsburgh, Pennsylvania (Becker); Department of Psychology, University of Pittsburgh School of Medicine, Pittsburgh, Pennsylvania (Becker); John P. Hussman Institute for Human Genomics, University of Miami, Miami, Florida (Beecham, Gilbert, Hamilton-Nelson, Martin, Perry, Wishnek); Dr John T. Macdonald Foundation Department of Human Genetics, University of Miami, Miami, Florida (Beecham, Gilbert, Martin); National Alzheimer’s Coordinating Center, University of Washington, Seattle (Beekly); Rush Alzheimer’s Disease Center, Rush University Medical Center, Chicago, Illinois (Bennett); Department of Pathology, Northwestern University Feinberg School of Medicine, Chicago, Illinois (Bigio); Cognitive Neurology and Alzheimer’s Disease Center, Northwestern University Feinberg School of Medicine, Chicago, Illinois (Bigio, Mesulam, Weintraub); Geriatric Research, Education, and Clinical Center, VA Puget Sound Healthcare System, Seattle, Washington (Bird, Tsuang); Department of Neurology, University of Washington, Seattle (Bird);

Department of Epidemiology, Harvard School of Public Health, Boston, Massachusetts (Blacker); Department of Psychiatry, Massachusetts General Hospital/Harvard Medical School, Boston (Blacker); Department of Neurology, Mayo Clinic, Rochester, Minnesota (Boeve, Petersen); Swedish Medical Center, Seattle, Washington (Bowen); Department of Neurology, University of California, San Francisco (Boxer, Karydas, B. L. Miller, Rosen, Seeley); Department of Medicine, Duke University, Durham, North Carolina (Burke, Welsh-Bohmer); Department of Neuroscience, Mount Sinai School of Medicine, New York, New York (Buxbaum); Department of Psychiatry, Mount Sinai School of Medicine, New York, New York (Buxbaum, Sano); Department of Genetics and Genomic Sciences, Mount Sinai School of Medicine, New York, New York (Buxbaum); Department of Pathology and Immunology, Washington University, St Louis, Missouri (Cairns, Morris); USF Health Byrd Alzheimer's Institute, University of South Florida, Tampa (Cao, Potter, Raj, Smith); Fred Hutchinson Cancer Research Center, Seattle, Washington (Carlson, McDavid); Department of Pathology, University of Alabama at Birmingham, Birmingham (Carroll, Fallon); Department of Neurology, University of Southern California, Los Angeles (Chui, L. S. Schneider); Department of Neurology, University of Alabama at Birmingham, Birmingham (Clark, Harrell, Marson, Roberson); Department of Neurology, University of California, Irvine (Cribbs, Pierce); Department of Psychiatry and Behavioral Sciences, Miller School of Medicine, University of Miami, Miami, Florida (Crocco); Department of Neurology, University of California, Davis, Sacramento (DeCarli, Olichney); University of Virginia School of Medicine, Charlottesville (DeKosky); now with University of Pittsburgh, Pittsburgh, Pennsylvania (DeKosky); Institute for Memory Impairments and Neurological Disorders, University of California, Irvine (Dick, Poon); Department of Neuroscience, Mayo Clinic, Jacksonville, Florida (Dickson, Ertekin-Taner, Graff-Radford, Younkin); Wien Center for Alzheimer's Disease and Memory Disorders, Mount Sinai Medical Center, Miami Beach, Florida (Duara); Department of Neurology, Mayo Clinic, Jacksonville, Florida (Ertekin-Taner, GraffRadford); Department of Neurology, Indiana University, Indianapolis (Farlow); Department of Psychiatry, New York University, New York (Ferris, Reisberg); C. S. Kubik Laboratory for Neuropathology, Massachusetts General Hospital, Charlestown (Frosch); Department of Neurosciences, University of California, San Diego, La Jolla (Galasko, Koo, Masliah);

Department of Pathology and Laboratory Medicine, Emory University, Atlanta, Georgia (Gearing); Alzheimer's Disease Research Center, Emory University, Atlanta, Georgia (Gearing); Neurogenetics Program, University of California, Los Angeles (Geschwind); Department of Pathology and Laboratory Medicine, Indiana University, Indianapolis (Ghetti, Murrell, Spina); Department of Neurology, Emory University, Atlanta, Georgia (Glass, Lah, Levey); Department of Neurology, Massachusetts General Hospital/Harvard Medical School, Boston (Growdon, Hyman, Tanzi); Division of Neuropathology, Department of Pathology, University of Pittsburgh, Pittsburgh, Pennsylvania (Hamilton); Department of Molecular and Biomedical Pharmacology, Sanders-Brown Center on Aging, University of Kentucky, Lexington (Head); Taub Institute for Research on Alzheimer's Disease and the Aging Brain, Columbia University, New York, New York (Honig, Reitz, Vardarajan, Vonsattel, Williamson); Department of Neurology, Columbia University, New York, New York (Honig, Reitz, Vardarajan); Department of Pathology, Duke University, Durham, North Carolina (Hulette); Department of Genome Sciences, University of Washington, Seattle (Jarvik); Division of Medical Genetics, Department of Medicine, University of Washington, Seattle (Jarvik); Department of Neurology, Sanders-Brown Center on Aging, University of Kentucky, Lexington (Jicha); Department of Pathology and Laboratory Medicine, University of California, Davis, Sacramento (Jin, J. W. Miller); Genetics Program, Department of Medicine, Boston University, Boston, Massachusetts (Jun); Department of Biostatistics, Boston University, Boston, Massachusetts (Jun, Lunetta); Department of Ophthalmology, Boston University, Boston, Massachusetts (Jun); Alzheimer Disease Research Center, University of Pittsburgh, Pittsburgh, Pennsylvania (Kamboh, Lopez); Department of Neurology, Oregon Health & Science University, Portland (Kaye, P. Kramer, Quinn); Department of Neurology, Portland Veterans Affairs Medical Center, Portland, Oregon (Kaye); Department of Pathology and Laboratory Medicine, University of California, Irvine (Kim); Department of Neurology, Boston University, Boston, Massachusetts (Kowall, McKee, Stern); Department of Pathology, Boston University, Boston, Massachusetts (Kowall, McKee); Department of Neuropsychology, University of California, San Francisco (J. H. Kramer); Department of Molecular and Medical Genetics, Oregon Health & Science University, Portland (P. Kramer); Department of Neurobiology and Behavior, University of California,

Irvine (LaFerla); Department of Pathology, University of Washington, Seattle (Leverenz, Montine, Sonnen); Department of Psychiatry and Behavioral Sciences, University of Washington School of Medicine, Seattle (Li, Peskind, Raskind, Tsuang); Department of Pathology, University of Michigan, Ann Arbor (Lieberman); Department of Psychiatry, Johns Hopkins University, Baltimore, Maryland (Lyketsos); Department of Preventive Medicine, University of Southern California, Los Angeles (Mack); Division of Pulmonary, Critical Care, and Sleep Medicine, Department of Medicine, New York University, New York (Martiniuk); Department of Neurology, University of Miami, Miami, Florida (Mash); Department of Pathology, University of California, San Diego, La Jolla (Masliah); Department of Medicine, University of Washington, Seattle (McCormick, C.-E. Yu); Northwest Research Group on Aging, School of Nursing, University of Washington, Seattle (McCurry); Department of Neurology, Northwestern University Feinberg School of Medicine, Chicago, Illinois (Mesulam); Department of Pathology, University of Southern California, Los Angeles (C. A. Miller); Department of Neurology, Washington University, St Louis, Missouri (Morris); Department of Medical and Molecular Genetics, Indiana University, Indianapolis (Murrell, Saykin); Department of Anatomic Pathology, Mayo Clinic, Rochester, Minnesota (Parisi); Department of Laboratory Medicine and Pathology, Mayo Clinic, Rochester, Minnesota (Parisi); Neurogenomics Division, Translational Genomics Research Institute, Phoenix, Arizona (Reiman); Arizona Alzheimer's Consortium, Phoenix (Reiman); Banner Alzheimer's Institute, Phoenix, Arizona (Reiman); Alzheimer's Disease Center, New York University, New York (Reisberg); Gertrude H. Sergievsky Center, Columbia University, New York, New York (Reitz, Vardarajan); Department of Neurology, University of Texas Southwestern Medical Center, Dallas (Rosenberg); Department of Radiology and Imaging Sciences, Indiana University, Indianapolis (Saykin); Department of Pathology (Neuropathology), Rush University Medical Center, Chicago, Illinois (J. A. Schneider); Department of Psychiatry, University of Southern California, Los Angeles (L. S. Schneider); Center for Human Genetics and Research, Department of Molecular Physiology and Biophysics, Vanderbilt University, Nashville, Tennessee (Thornton-Wells); Department of Pathology and Laboratory Medicine, University of Pennsylvania Perelman School of Medicine, Philadelphia (Trojanowski, Van Deerlin); Department of Pathology, Johns Hopkins University, Baltimore, Maryland

(Troncoso); Department of Anatomy and Neurobiology, Sanders-Brown Center on Aging, University of Kentucky, Lexington (Van Eldik); Department of Pathology and Laboratory Medicine, University of California, Los Angeles (Vinters); Department of Pathology, Columbia University, New York, New York (Vonsattel); Department of Psychiatry, Northwestern University Feinberg School of Medicine, Chicago, Illinois (Weintraub); Department of Psychiatry and Behavioral Sciences, Duke University, Durham, North Carolina (Welsh-Bohmer); Department of Pathology, Oregon Health & Science University, Portland (Woltjer); Evelyn F. McKnight Brain Institute, Department of Neurology, Miller School of Medicine, University of Miami, Miami, Florida (Wright).

Conflict of Interest Disclosures

Drs Graham and Behrens are full-time employees of Genentech, Inc. Dr DeKosky is a consultant for AstraZeneca, Eli Lilly and Co, Merck and Co, and Rivermend and is involved in clinical trials with Baxter, Elan, Janssen, Novartis, and Pfizer. Dr Farlow receives research support from Accera, Biogen, Eisai, Eli Lilly and Co, Genentech, MedAvante/ AstraZeneca, and Navidea; is on the speaker's bureau at Eisai, Pfizer, Forest, Novartis, and Eli Lilly and Co; and is a consultant or advisory board member for Accera, Alltech, Avanir, Eisai, Med Res, Hilicon, Medavante, Medivation, Merck and Co, Novartis, Pfizer, Prana Biotech, QR Pharma, Roche, Sanofi-Aventis, Schering-Plough, Toyama Pharm, Eli Lilly and Co, and UCB Pharma. Dr Ghetti has consulted for Piramal Imaging. Dr Leverenz is a consultant for Boehringer-Ingelheim, Navidea Biopharmaceuticals, and Piramal Healthcare. Dr Petersen is chair for the data monitoring committee for Pfizer and Janssen Alzheimer Immunotherapy and is a consultant for GE Healthcare and Roche. Dr Wright receives royalties from UpTo Date for 2 chapters; has done legal consulting for the law firms of Abali, Milne, and Faegre Baker Daniels; is a consultant for Merck and Co; and does stroke adjudication for a National Institutes of Health clinical trial. No other disclosures were reported.

Funding/Support

The National Institute on Aging supported this work through the following grants: U01 AG032984 and RC2 AG036528 (Alzheimer's Disease Genetics Consortium); NIAGADS U24-AG041689; U01 AG016976 (National Alzheimer's Coordinating Center); U24 AG021886 (National Cell Repository for Alzheimer's Disease); U24 AG026395, R01 AG041797, and U24 AG026390 (National Institute on Aging–Late-Onset Alzheimer's Disease); P30 AG019610 (Banner Sun Health Research Institute); P30 AG013846, R01 HG02213, K24 AG027841, U01 AG10483, R01 CA129769, and R01 MH080295 (Boston University); P50 AG008702 (Columbia University); P30 AG028377 (Duke University); AG025688 (Emory University); P30 AG10133 (Indiana University); P50 AG005146 (Johns Hopkins University); P50 AG005134 (Massachusetts General Hospital); P50 AG016574 (Mayo Clinic); P50 AG005138 (Mount Sinai School of Medicine); P30 AG08051, U01 AG16976, MO1 RR00096, and UL1 RR029893 (New York University); P30 AG013854 (Northwestern University); P30 AG008017 (Oregon Health & Science University); P30 AG010161 (Rush University); P50 AG016582 and UL1 RR02777 (Center for Clinical and Translational Science, University of Alabama at Birmingham); P30 AG010129 (University of California, Davis); P50 AG016573, P50 AG016574, P50 AG016575, P50 AG016576, and P50 AG016577 (University of California, Irvine); P50 AG016570 (University of California, Los Angeles); P50 AG005131 (University of California, San Diego); P50 AG023501 and P01 AG019724 (University of California, San Francisco); P30 AG028383 (University of Kentucky); P50 AG008671 (University of Michigan); P30 AG010124 (University of Pennsylvania); P50 AG005133 (University of Pittsburgh); P50 AG005142 (University of Southern California); P30 AG012300 (University of Texas Southwestern Medical Center); P50 AG005136 and R01 AG007584 (University of Washington); and P50 AG005681 and P01 AG03991 (Washington University). The Alzheimer's Disease Genetics Consortium is also supported by a grant from a private foundation wishing to remain anonymous. The work completed by Boston University is also supported by grant IIRG-08-89720 from the Alzheimer's Association and by the VA New England Geriatric Research Education and Clinical Center. The work by Miami University is also supported by grant R01 AG027944 from the National Institute on Aging and by the Alzheimer's Association. This project was also made possible by the many contributions of individual study data sets, supported in part by the National Institutes of Health, including Adult Changes in Thought and

Electronic Medical Records and Genetics (eMERGE) (grants U01 AG06781, U01 HG004610, and U01 HG006375 from the National Human Genome Research Institute), Columbia University Washington Heights–Inwood Columbia Aging Project (R01 AG037212 from the National Institutes of Health), and MIRAGE (R01 AG009029 from the National Institute on Aging). The University of Miami/Vanderbilt University/Mount Sinai School of Medicine work was supported by grants AG010491, AG002219, AG005138, AG027944, AG021547, AG019757, and R01 AG027944 from the National Institute on Aging and IIRG-05-14147 from the Alzheimer's Association. A subset of these participants was ascertained while Dr Pericak-Vance was a faculty member at Duke University. Clinical data and genotyping efforts for the Religious Orders Study and the Memory and Aging Project were supported by grants P30 AG10161, R01 AG15819, R01 AG17917, and R01 AG30146 from the National Institute on Aging and by the Illinois Department of Public Health and the Translational Genomics Research Institute. Data and samples from the National Institute on Aging–Late Onset Alzheimer's Disease Family Study, which receives government support under cooperative agreement grant U24AG026390 from the National Institute on Aging, were used in this study. Data from the Cache County Study on Memory Health and Aging were supported by grants R01 AG042611 from the National Institute on Aging and MNIRG-11-205368 from the Alzheimer's Association and by the Brigham Young University Gerontology Program and the Charleston Conference on Alzheimer's Disease to Brigham Young University; they were also supported by grants R01 AG11380, R01 AG21136, and R01AG3272 from the National Institutes of Health and by the Utah Science, Technology, and Research Initiative and the Utah State University Agricultural Experiment Station to Utah State University. Samples from the National Cell Repository for Alzheimer's Disease, which receives government support under cooperative agreement grant U24 AG21886 from the National Institute on Aging, were used in this study. Dr St George-Hyslop is supported by the Wellcome Trust, Howard Hughes Medical Institute, and Canadian Institute of Health Research. Drs Barmada, Demirci, and Kamboh are supported by grant AG030653 from the National Institute on Aging. Dr Wright is supported by grants K02 NS 059729 from the National Institute of Neurological Disorders and Stroke, R01 HL108623 from the National Heart, Lung, and Blood Institute, and SDG 0735387N from the American Heart Association.

Group Information

The National Institute on Aging–Late-Onset Alzheimer's Disease (NIA-LOAD) Family Study investigators include the following: Boston University, Boston, Massachusetts: Robert C. Green, MD, MPH, Neil W. Kowall, MD, Lindsay A. Farrer, PhD; Columbia University, New York, New York: Jennifer Williamson, MS, MPH, Vincent Santana, MBA; Duke University, Durham, North Carolina: Donald Schmechel, MD, Perry Gaskell, BS, Kathleen A. Welsh-Bohmer, PhD; Indiana University, Indianapolis: Bernardino Ghetti, MD, Martin R. Farlow, MD, Kelly Horner; Massachusetts General Hospital, Boston: John H. Growdon, MD, Deborah Blacker, MD, ScD, Rudolph E. Tanzi, PhD, Bradley T. Hyman, MD, PhD; Mayo Clinic, Rochester, Minnesota: Bradley F. Boeve, MD, Karen Kuntz, RN, Lindsay Norgaard, BS, Nathan Larson, BS; Mayo Clinic, Jacksonville, Florida: Dana Kistler, BSH, Francine Parfitt, MS, Jenny Haddow, BS; Mount Sinai School of Medicine, New York, New York: Jeremy Silverman, PhD, Michal Schnaider Beeri, PhD, Mary Sano, PhD, Joy Wang, BA, Rachel Lally, BA; Northwestern University, Chicago, Illinois: Nancy Johnson, PhD, M. Marsel Mesulam, MD, Sandra Weintraub, PhD, Eileen H. Bigio, MD; Oregon Health & Science University, Portland: Jeffery A. Kaye, MD, Patricia Kramer, PhD, Jessica Payne-Murphy, BA; Rush University, Chicago, Illinois: David A. Bennett, MD, Holli Jacobs, BA, Jeen-Soo Chang, MD, Danielle Arends, RN; University of Alabama at Birmingham, Birmingham: Lindy E. Harrell, MD, PhD; University of California, Los Angeles: George Bartzokis, MD, Jeffery Cummings, MD, Po H. Lu, PsyD, Usha Toland, MS; University of Kentucky, Lexington: William Markesbery, MD, Charles Smith, MD, Alise Brickhouse, BA; University of Pennsylvania, Philadelphia: Gerard D. Schellenberg, PhD, John Q. Trojanowski, MD, PhD, Vivianna M. Van Deerlin, MD, PhD, Elisabeth McCarty Wood, MS; University of Pittsburgh, Pittsburgh, Pennsylvania: Steven T. DeKosky, MD, Robert Sweet, MD, Elise Weamer, MPH; University of Southern California, Los Angeles: Helena C. Chui, MD, Arousiak Varpetian, MD; University of Texas Southwestern Medical Center, Dallas: Ramon Diaz-Arrastia, MD, PhD, Roger N. Rosenberg, MD, Barbara Davis, MA; University of Washington, Seattle: Thomas D. Bird, MD, Malia Rumbaugh, MS, Murray Raskind, MD; Washington

University, St Louis, Missouri: Alison M. Goate, DPhil, John C. Morris, MD, Joanne Norton, MSN, RN, Denise Levitch, RN, Betsy Grant, MSW, PhD, Mary Coats, MSN, RN.

Additional Contributions

Creighton Phelps, PhD, Stephen Snyder, PhD, and Marilyn Miller, PhD, National Institute on Aging, Bethesda, Maryland, helped in acquiring samples and data and are ex officio members of the Alzheimer's Disease Genetics Consortium. We thank the contributors, including the Alzheimer's Disease Centers who collected samples used in this study, as well as the patients and their families, whose help and participation made this work possible.
