# Supplementary figures and images for: Caspase-8, association with Alzheimer’s Disease and functional analysis of rare variants
Source: PLoS One. 2017 Oct 6;12(10):e0185777. doi: 10.1371/journal.pone.0185777 (PMC5630132; doi:10.1371/journal.pone.0185777)

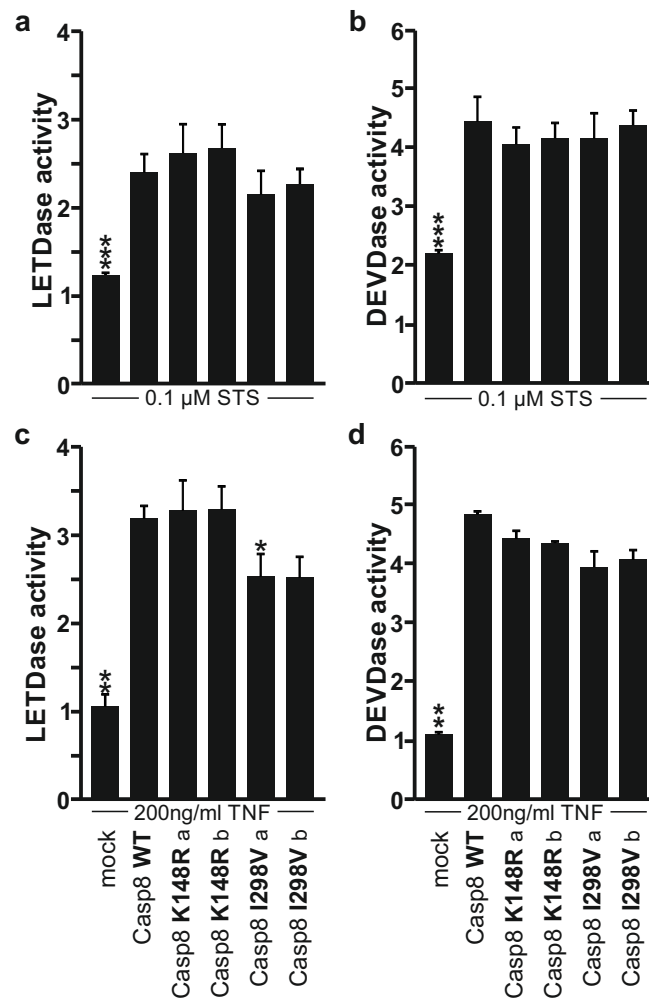

supplementary Figure 1. Rehker J, Rodhe J, et al.

Supplement: S1 Fig — (a to d) SK-N-BE(2) cells were transfected with expression vector encoding Caspase-8 WT, Caspase-8 K148R, or Caspase-8 I298V and mock as control. Twenty-four hours post-transfection, cells were treated with 0.1μM staurosporine (STS) (a, b) or 200ng/ml tumor necrosis factor (TNF) (c, d) for an additional 6 hours. Thereafter, caspase-8-related LETDase activity (a, c), and downstream Caspase-3-like-related DEVDase activity (b, d) were monitored. Data are presented as fold over mock untreated. Statistics and error bars: mean±s.d. n = 3–5 of biological replicates. Data was analyzed as comparison to Caspase-8 WT using two-sided student’s t-test. *P< 0.05; **P< 0.01 and ***P< 0.001. (PDF) [file pone.0185777.s004.pdf]

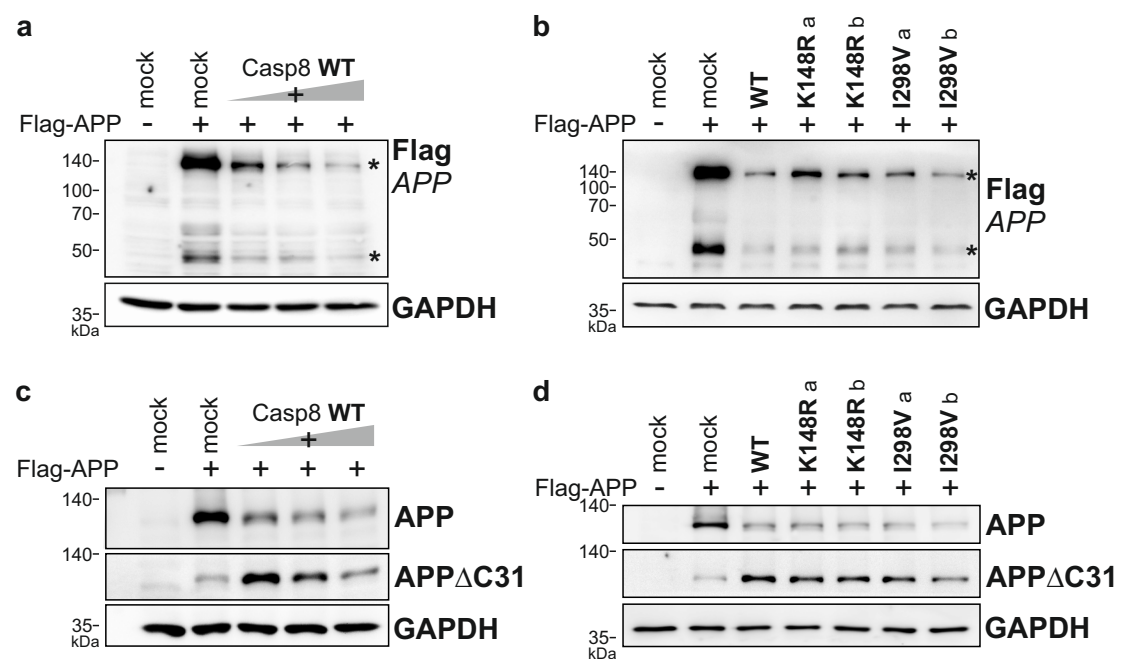

Supplementary Figure 2. Rehker J, *et al.*

Supplement: S2 Fig — (a to d) SK-N-BE(2) cells were co-transfected with expression vector encoding Flag tagged APP together plasmid for Caspase-8 WT, Caspase-8 K148R, or Caspase-8 I298V and mock as control. Caspase-8 WT expression lead to dose-dependent processing of Flag-APP as detected with antibodies directed against the Flag-tag epitope (a) or the APP protein itself (c). The Caspase-8 K148R and Caspase-8 I298V mutants are also able to cleave APP, as indicated by the reduced levels of APP detected with anti-Flag (b) or anti-APP antibodies (d). Introduction of Caspase-8 WT leads to cleavage of APP at its VEVD664 caspase-cleavage site, resulting in the formation of an APP ΔC31 fragment (c). Elevated levels of APP ΔC31 can be detected in Caspase-8 WT as well as the Caspase-8 K148R and Caspase-8 I298V transfected cells (d). *indicates APP and cleaved APP detected by Flag antibody. (PDF) [file pone.0185777.s005.pdf]
